# Unraveling the glycosphingolipid metabolism by leveraging transcriptome-weighted network analysis on neuroblastic tumors

**DOI:** 10.1186/s40170-024-00358-y

**Published:** 2024-10-24

**Authors:** Arsenij Ustjanzew, Annekathrin Silvia Nedwed, Roger Sandhoff, Jörg Faber, Federico Marini, Claudia Paret

**Affiliations:** 1 Institute of Medical Biostatistics, Epidemiology and Informatics (IMBEI), University Medical Center of the Johannes Gutenberg-University Mainz, Mainz, 55131 Germany; 2 Lipid Pathobiochemistry, German Cancer Research Center, Heidelberg, 69120 Germany; 3 Department of Pediatric Hematology/Oncology, Center for Pediatric and Adolescent Medicine, University Medical Center of the Johannes Gutenberg-University Mainz, Mainz, 55131 Germany; 4 University Cancer Center (UCT), University Medical Center of the Johannes Gutenberg-University Mainz, Mainz, 55131 Germany; 5 Research Center for Immunotherapy (FZI), Mainz, 55131 Germany

**Keywords:** Neuroblastoma, Ganglioneuroma, Ganglioneuroblastoma, Ganglioside, GD2, Glycosphingolipids, Reaction activity score, Metabolic graph

## Abstract

**Background:**

Glycosphingolipids (GSLs) are membrane lipids composed of a ceramide backbone linked to a glycan moiety. Ganglioside biosynthesis is a part of the GSL metabolism, which involves sequential reactions catalyzed by specific enzymes that in part have a poor substrate specificity. GSLs are deregulated in cancer, thus playing a role as potential biomarkers for personalized therapy or subtype classification. However, the analysis of GSL profiles is complex and requires dedicated technologies, that are currently not included in the commonly utilized high-throughput assays adopted in contexts such as molecular tumor boards.

**Methods:**

In this study, we developed a method to discriminate the enzyme activity among the four series of the ganglioside metabolism pathway by incorporating transcriptome data and topological information of the metabolic network. We introduced three adjustment options for reaction activity scores (RAS) and demonstrated their application in both exploratory and comparative analyses by applying the method on neuroblastic tumors (NTs), encompassing neuroblastoma (NB), ganglioneuroblastoma (GNB), and ganglioneuroma (GN). Furthermore, we interpreted the results in the context of earlier published GSL measurements in the same tumors.

**Results:**

By adjusting RAS values using a weighting scheme based on network topology and transition probabilities (TPs), the individual series of ganglioside metabolism can be differentiated, enabling a refined analysis of the GSL profile in NT entities. Notably, the adjustment method we propose reveals the differential engagement of the ganglioside series between NB and GNB. Moreover, *MYCN* gene expression, a well-known prognostic marker in NTs, appears to correlate with the expression of therapeutically relevant gangliosides, such as GD2. Using unsupervised learning, we identified subclusters within NB based on altered GSL metabolism.

**Conclusion:**

Our study demonstrates the utility of adjusting RAS values in discriminating ganglioside metabolism subtypes, highlighting the potential for identifying novel cancer subgroups based on sphingolipid profiles. These findings contribute to a better understanding of ganglioside dysregulation in NT and may have implications for stratification and targeted therapeutic strategies in these tumors and other tumor entities with a deregulated GSL metabolism.

**Supplementary Information:**

The online version contains supplementary material available at 10.1186/s40170-024-00358-y.

## Background

**Fig. 1:**
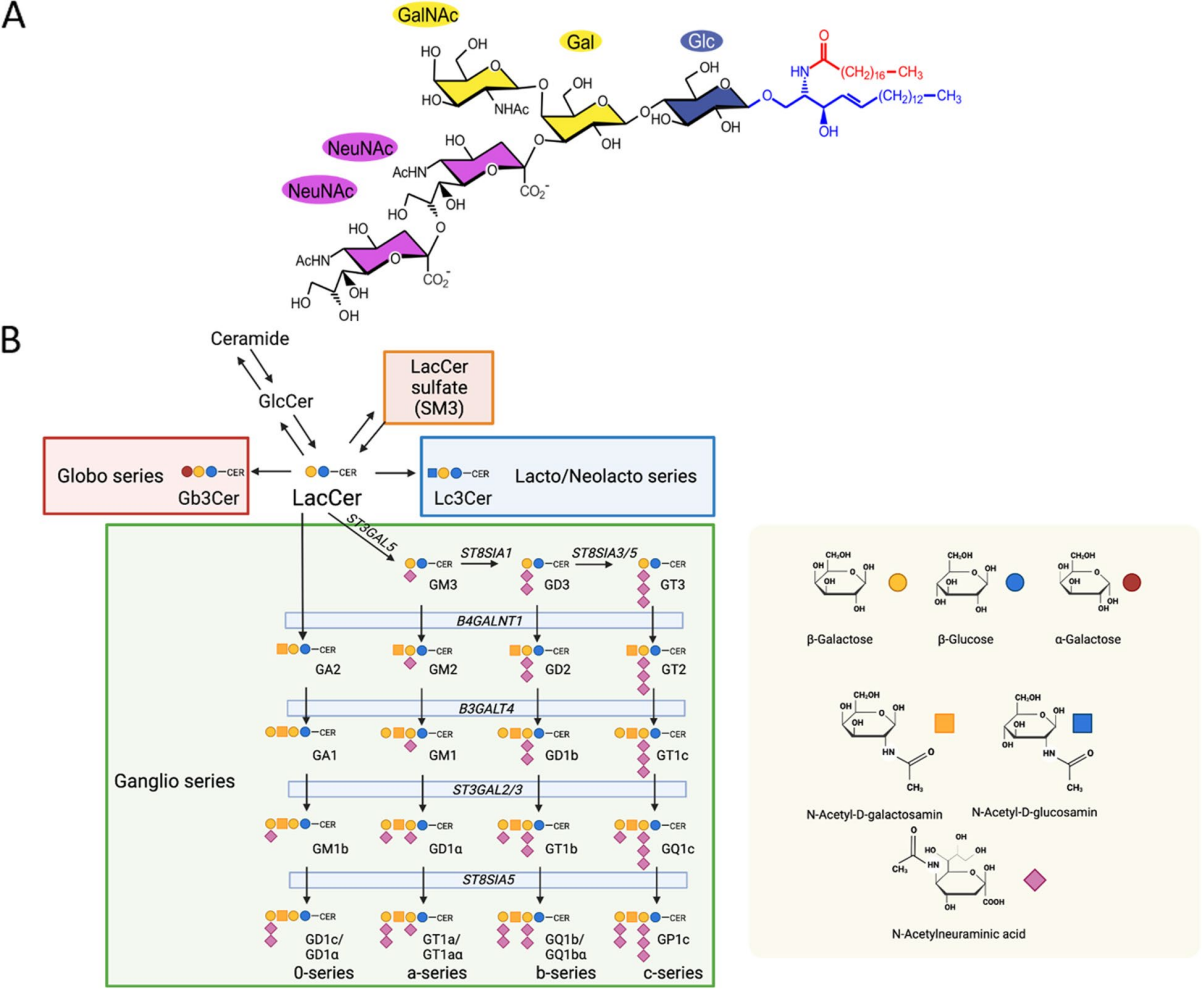
Molecular structure of disialoganglioside GD2 and the GSL biosynthesis pathway with focus on ganglioside biosynthesis. A) Exemplarily, GD2’s head group consists of $\beta$-Glucose (Glc), linked to the ceramide backbone, $\beta$-galactose (Gal), *N*-acetyl-D-galactosamin (GalNAc), and two *N*-acetylneuraminic acid molecules (NeuNAc). The sphingoid base and the fatty acid of the ceramide anchor are respectively blue and red. B) The GSL biosynthesis pathway starts with the synthesis of glucosylceramide (GlcCer) from ceramide and the further synthesis from GlcCer to lactosylceramide (LacCer). The addition of sulfate groups to LacCer leads to lactosylceramide sulfate (SM3). From LacCer the biosynthesis pathway splits into three subseries, the globo series, the lacto- and neolacto series, and the ganglio series. The ganglio-series consists of four subseries, the 0-, a-, b-, and c-series. The genes coding for the enzymes responsible for the ganglioside biosynthesis are *B4GALNT1*, *B3GALT4*, *ST3GAL2/3/5*, and *ST8SIA1/3/5*. Figure elements created with Biorender

Gangliosides are sialylated glycosphingolipids (GSLs) found in high concentration in the nervous system [1, 2]. Gangliosides are composed of a lipid anchor, consisting of sphingosine, a fatty acid of different lengths, and a polar head group composed of sialylated oligosaccharides (Fig. 1A) [1, 2]. The GSL biosynthesis pathway, shown in Fig. 1B, is a complex series of coordinated reactions occurring in the Golgi apparatus [3, 4]. Early steps in this pathway, which guide into a-, b-, and c-series are performed with enzymes of relative high substrate specificity, whereas downstream enzymes are promiscuitive and elongate all four series [5]. This circumstance results in the fact that the same enzymes are involved in reactions of all four series, for example, the protein encoded by *B3GALT4* converts GA2, GM2, GD2, and GT2 into the more complex gangliosides GA1, GM1, GD1b, and GT1c, respectively. Further, the pattern of ganglioside expression changes during the development in normal brain tissues. Simple gangliosides such as GD2 are expressed in the embryo, while mature neurons express complex gangliosides of the a-, and b-series, namely GM1a, GD1a, GD1b, and GT1b [6–9].

GSL profiles are often found to be deregulated in tumors [10, 11]. These changes have been particularly studied in neuroblastic tumors (NT) [12–15]. NTs are the most common extra-cranial solid tumors in children and include neuroblastoma (NB), ganglioneuroblastoma (GNB), and ganglioneuroma (GN) [16–18]. They arise from primitive neuroectodermal cells of the neural crest, which give rise to the sympathetic nervous system during the embryonic development [18]. One prominent feature of NTs is their biological and clinical heterogeneity. NT range from immature, undifferentiated to mature, differentiated tumors, where GNB and GN represent the mature end of this range [18, 19]. NBs often express simple gangliosides, particularly GD2, lacking or having reduced concentrations of the more complex a-, and b-series gangliosides. More mature NT such as GNB express very low amounts of GD2 and show instead an increased level of complex gangliosides of the a- and b-series [15, 20].

Ganglioside profiles in NB have been linked to prognosis and are relevant for therapy. Patients with tumors expressing higher concentrations of the b-series gangliosides, GD1b, and GT1b have a better prognosis [21]. The ganglioside GD2 is used as a target for the treatment of high-risk NB with monoclonal antibodies (Dinutuximab and Naxitamab, both already used in clinical protocols) and CAR-T cells (in clinical studies for several tumor entities) [22–24].

The simple gangliosides GD3 and GD2 regulate several receptors and signaling cascades and contribute to the undifferentiated state of neural stem cells and NB [25]. Discordant hypotheses exist on the mechanism of GD2 accumulation, with some reports claiming a low expression of *ST8SIA1* (required for the synthesis of GD3) and others a high expression of the same gene as the reason for GD2 expression [26, 27].

To understand and exploit GSLs, a better characterization of their molecular profiles is necessary. However, the measurement of the different GSLs is complex and requires dedicated protocols and instruments such as thin-layer chromatography and mass spectrometry [15]. RNA sequencing (RNA-seq) data obtained from patient-derived samples, on the other hand, provides a powerful opportunity for studying the expression of the genes required for ganglioside synthesis, as it is frequently collected during high-dimensional molecular cancer profiling, such as in the context of a Molecular Tumor Board [28]. However, the translation of transcriptome data into ganglioside profiles is challenging, and few models have been proposed so far to predict the expression of selected gangliosides from RNA-seq data [29–31].

In the following text, the terms network and graph are used interchangeably. The GSL biosynthesis pathway can be studied as a metabolite-centric network, where nodes are metabolites and edges represent the metabolic reactions. The edges can be weighted by the reaction activity scores (RAS) (first introduced by Graudenzi A. et al. (2018)), which can be considered as measurement of the activity of each reaction in the metabolic graph and is calculated using the normalized gene expression levels of a patient sample and the gene-protein-reaction (GPR) association rules of a genome-scale metabolic model [32–34]. As a result of the low specificity of the involved enzymes, the reaction activity cannot be distinguished across the four series by simply weighting edges of the network by the respective RAS values. This prevents the analysis of a cell type-specific GSL profile based on transcriptional regulation and hinders a more detailed understanding of the underlying biological processes and functions associated with the ganglioside metabolism pathway.

In this study, our primary objectives were 1) to develop a method based on RNA-seq data that can discriminate between the four series of the ganglioside metabolism pathway, 2) to test the method on NT entities, and finally 3) to interpret the results in the context of published ganglioside measurements. We hereby introduced three adjustment methods for the RAS by incorporating the network topology and its transition probabilities (TPs) across the four series. We demonstrated how these weighting schemes can be used in 1) an exploratory, unsupervised manner, and 2) in the direct comparison between several defined groups by applying our approach on two NT datasets. On the one hand, our approach enhances our comprehension of GSL deregulation in NT, and its transferability suggests potential application to other tumor entities, prospectively leading to the development of new pre-screening tools for assessing GSL metabolism in patients. On the other hand, it is conceivable that the method applied here may be transferable to other metabolic pathways involving non-specific enzymes, broadening its utility beyond the scope of ganglioside metabolism.

## Methods

### Workflow

**Fig. 2:**
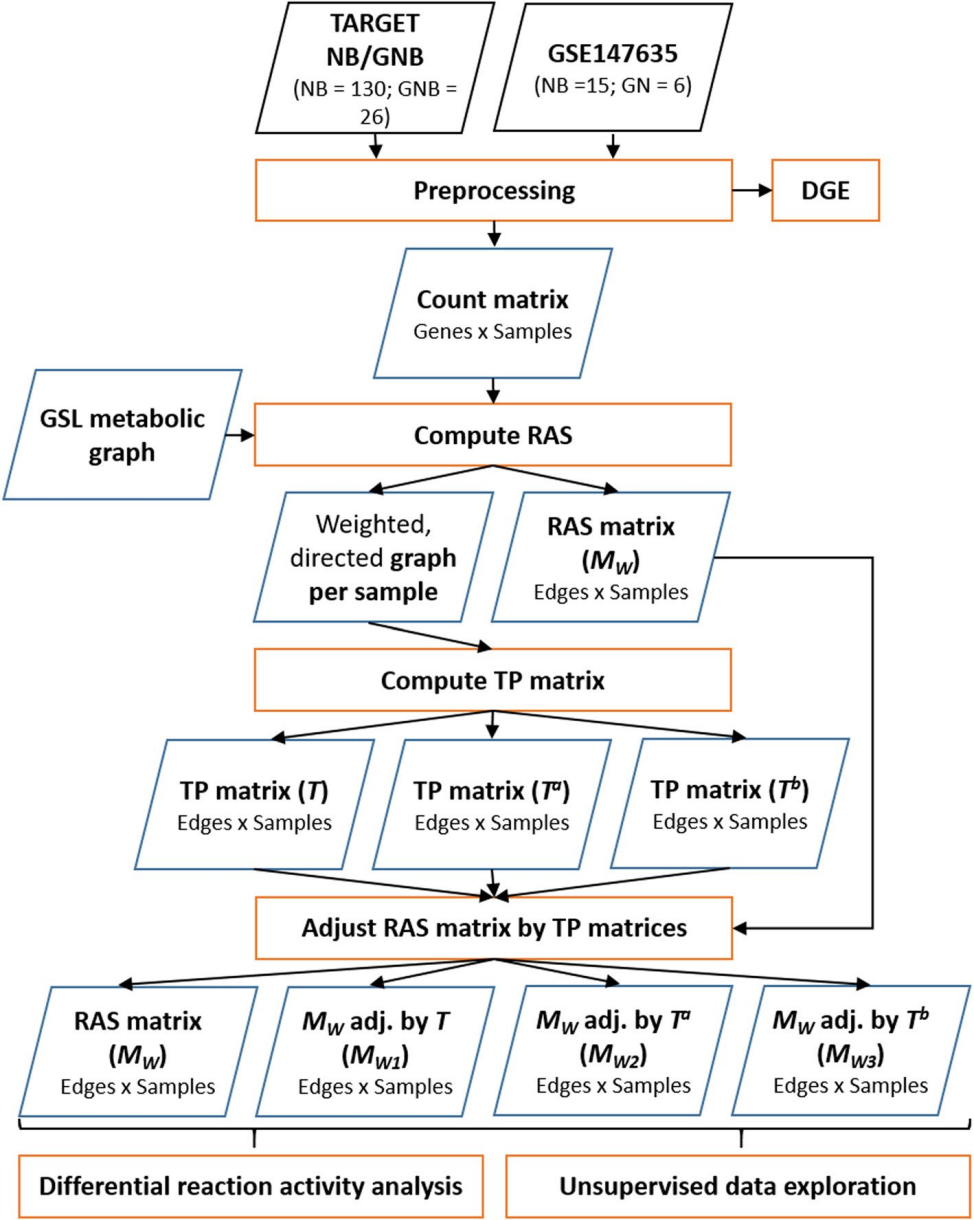
Workflow diagram representing the data processing steps (orange rectangles), the input data (black parallelograms), and the intermediate outputs (blue parallelograms). NB=Neuroblastoma; GNB=Ganglioneuroblastoma; GN=Ganglioneuroma; DGE=Differential gene expression; GSL=Glycosphingolipid; RAS=Reaction activity score; TP=Transition probability

We followed the workflow schematically illustrated in Fig. 2. Two publicly accessible RNA-seq datasets containing NT samples were acquired. The analysis workflow was conducted separately for each dataset. Both datasets were preprocessed by data normalization and log10 transformation with one pseudocount. The resulting normalized count matrices were used for further processing steps. A GSL metabolic graph was constructed (see further details in “Graph construction” section below), representing the reactions between metabolites and genes in the dataset. The RAS for each reaction of the metabolic graph were computed resulting in a weighted, directed graph per sample and a RAS matrix over all samples.

A transition probability (TP) matrix was computed for each weighted graph, describing the probability of moving from one node to another in the metabolic pathway based on the RAS values of the outgoing edges. Two alternative versions of the TP matrix were also computed to address the discrimination of the ganglioside 0-, a-, b-, and c-series (see subsection “Alternative transition probability matrices”). Finally, the RAS matrix was adjusted using the transition matrices, resulting in four distinct matrices.

The (adjusted) RAS matrices were further used for (1) unsupervised data exploration, and (2) differential reaction analysis between groups. For the first approach, unsupervised machine learning methods were used for exploratory analysis of the adjusted RAS values. This includes dimensionality reduction with UMAP, identification of clusters of samples based on their RAS profiles, and the identification of statistically differential expressed reactions between clusters. In the differential reaction analysis, the dataset-specific groups were compared by computing a log2 fold-change of the RAS values. *P*-values per reaction were determined by using the Kolmogorov-Smirnov (KS) test.

The workflow was executed entirely in the R environment (version 4.1.3). Box plots, dot plots and scatter plots were generated with ggplot2 (version 3.4.2) [35] and plotly (version 4.10.1). Heatmaps were drawn with the pheatmap library (version 1.0.12). The networks were visualized with the R package igraph (version 1.4.2) [36].

### Data acquisition and preprocessing

Two datasets were used:

(1) Expression data and corresponding clinical information were obtained from GSE147635 [37] (n=21). This dataset contains 6 GN and 15 NB samples. The RASflow pipeline was used as the framework for the quantification of gene expression [38]. HISAT2 tool [39] in combination with HTSeq [40] was used on the human genome GRCh38 (GCA_000001405.15) and the GENCODE annotation (version 31) [41] to retrieve gene counts. In the further course of the text this dataset is called GN/NB dataset.

(2) The Therapeutically Applicable Research to Generate Effective Treatments (TARGET) NB gene-expression profile (“RSEM expected_count”) and clinical data (*n* = 162) were acquired from the UCSC Xena database (http://xena.ucsc.edu/) [39] as $log2(expected\_count+1)$. The gene counts were back-transformed to integer counts. This dataset contains 26 GNB and 130 NB. Six samples of unknown subtypes were excluded. Further, this dataset contains the “MYCN.status” variable, resulting in 33 MYCN-amplified (+MYCN), 128 without a MYCN amplification (-MYCN), and one sample with unknown MYCN amplification status. This one sample of unknown status was removed. A GNB sample has the annotation stating that it is MYCN-amplified. A MYCN amplification is rare in GNB, so we also removed it from the dataset due to lack of power as it was detected in only one sample. The final dataset remained 154 samples in total. In the following, we refer to this dataset as the TARGET GNB/NB dataset.

All datasets were separately normalized by the median of ratios method using the DESeq2 R package (version 1.34.0) [42]. A pseudocount was added to the counts and then log10 transformed. For the distance heatmaps of both datasets, the raw counts were normalized and a variance stabilizing transformation was applied using the vst function of DESeq2.

### Graph construction

A metabolite-centric graph, where nodes are metabolites and edges connect the metabolites involved in the same reactions, was built with the R packages NetPathMiner (version 1.30.0) [43] and igraph (version 1.4.2) [36]. The metadata of the edges contains the involved enzymes. The graph consists of the four pathways (hsa00600, hsa00601, hsa00603, hsa00604) from the Kyoto Encyclopedia of Genes and Genomes (KEGG) [44] (the KGML files were retrieved on 07/07/2022) and represents the sum of sphingolipid & glycosphingolipid metabolism including the lacto-, neolacto-, globo-, and ganglio series. The reactions R06010 and R06004 representing the degradation of GM1 to GM2 and GM2 to GM3 were removed from the graph. This is justified because these reactions represent the degradation process and are not competing enzymatic processes of the biosynthesis. The resulting metabolic graph is used in later steps as a template graph. Additionally, most enzymes in the ganglioside biosynthesis pathway are exclusively involved in the GSL pathways [45]. Out of the 90 considered enzymes, 56 are also involved in non-GSL pathways, which are often related to lipids or glycans, such as the Sphingolipid signaling pathway, Lysosome, and Ether lipid metabolism. The final graph annotation can be seen in Fig. S1 and Additional file 2 and an igraph object can be found at https://github.com/arsenij-ust/NT_GSL_analysis.

### Weighting of the metabolic graph

#### Computing RAS values

**Fig. 3:**
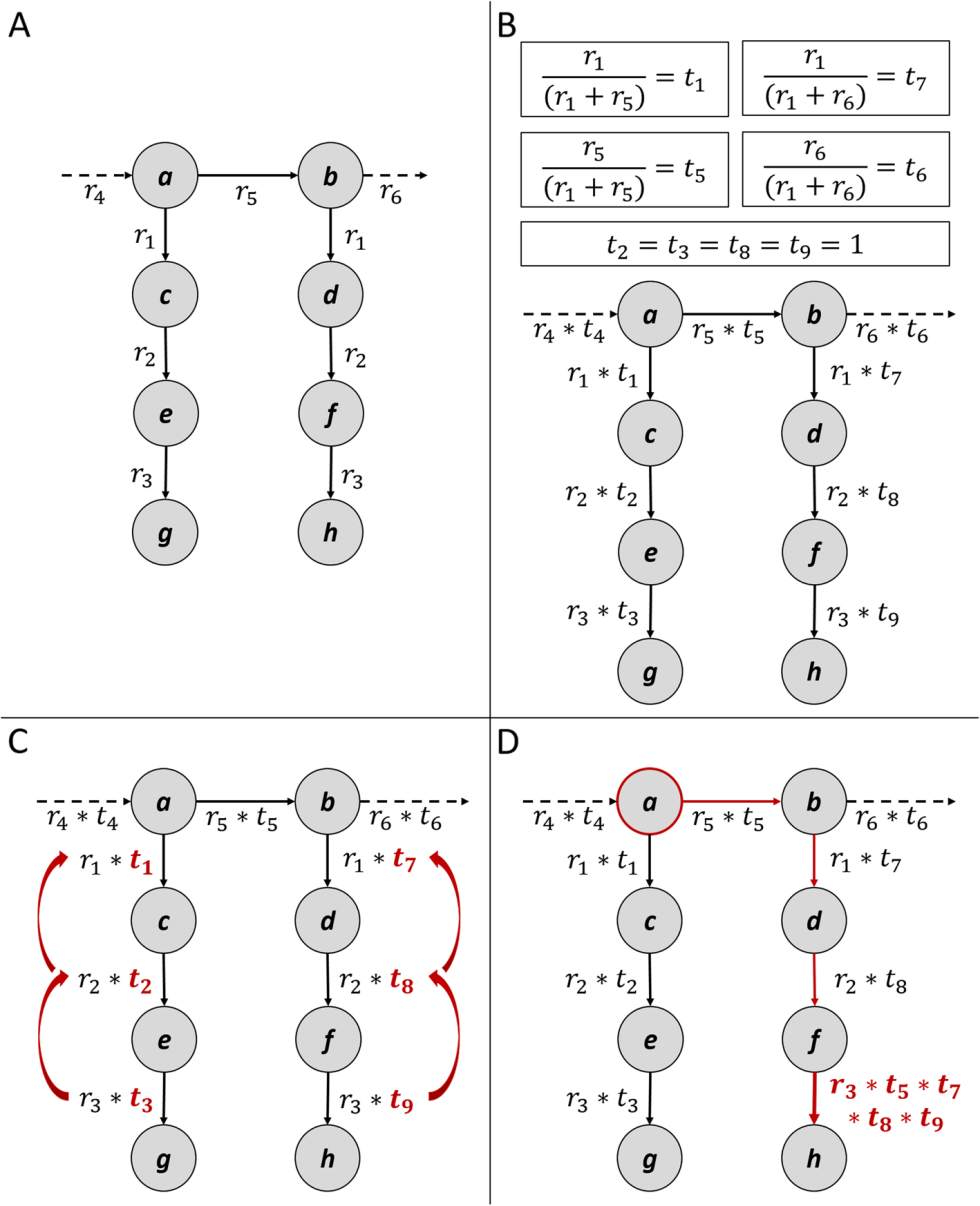
Visual examples of introduced adjustment methods based on transition probability. **A** An exemplary graph, where nodes represent metabolites (*a* - *h*) and edges are RAS values of the respective reactions (*r*). Although formally the reactions from nodes *a* to *c* and *b* to *d* are separate reactions with distinct reaction IDs, the RAS values of these reactions are identical because of the involvement of the exact same genes. Therefore, we denote these reactions with identical *r*-numbers ($r_{1}$) in this figure. The same also applies to edges $r_{2}$ and $r_{3}$. Ingoing and outgoing edges are dashed. **B** Edge weights are adjusted by multiplying the TP *t* with the RAS value of the respective reaction *r*. Exemplary calculations of the TPs are given for $t_{1}$, $t_{5}$, $t_{6}$, and $t_{7}$. The TP of $t_{2}$, $t_{3}$, $t_{8}$, and $t_{9}$ are equal to 1 because nodes *c*, *d*, *e*, and *f* have only one outgoing edge. **C** First alternative adjustment method, in which the TP is equal to 1, recursively takes the TP of the previously incoming edges. **D** Second alternative adjustment method, where exemplary the RAS value $r_{3}$ is adjusted by the product of TPs along the path starting from the defined node *a* to node *h*

The normalized gene expression values $C_{i,j}$ of a patient sample in the form of an $n \times m$ matrix *C*, where *n* is the number of genes and *m* is the number of samples, are used to compute the RAS. RAS defines the amount of activity in a certain condition, for each reaction of the metabolic graph (Fig. 3A). RAS was computed as described in Graudenzi A. et al. [32] based on the GPR association rules of the genome-scale metabolic model of the Homo sapiens (Human1) [46]. GPRs are logical formulas explaining the association between gene products in the process of catalyzation of a given reaction. These formulas involve logical operators, such as AND and OR. Thus, for each sample $s = 1,...n$, and each reaction *r* the RAS is computed based on the following distinctions:

1) Reactions with AND operator (i.e., enzyme subunits):

1 $$\begin{aligned} RAS_{s,r} = min(C_{i,j}:U_{r}) \end{aligned}$$

where $U_{r}$ is the set of genes that encode the subunits of the enzyme catalyzing reaction *r*.

2) Reactions with OR operator (i.e., enzyme isoforms):

2 $$\begin{aligned} RAS_{s,r} = \sum \limits _{i\in I_{r}} \end{aligned}$$

where $I_{r}$ is the set of genes that encode isoforms of the enzyme that catalyzes reaction *r*.

RAS values of a sample are used to weight the associated edges, resulting in a weighted directed graph $G=(V, E, W)$, where *V* and *E* are the set of all nodes (metabolites) and edges (reactions) in *G*, and *W* is the weighted adjacency matrix containing the RAS values. Our resulting metabolic graph contains 116 reactions, of which two reactions have AND logic, 51 reactions have OR logic, and 63 reactions have only one assigned gene.

#### Computing the transition probability matrix

Let $G_1, G_2, \dots , G_s$ be a list of *s* graphs (one graph per sample), where each graph $G_i=(V, E, W)$ has an associated transition matrix $T_i$. $T_i$ is computed as follows:

Let $W_i$ be the $n_i \times n_i$ weighted adjacency matrix for graph $G_i$, where the element $w_{i,j}$ represents the RAS value of the reaction catalyzed from node *i* to node *j* in graph $G_i$.

The row sums of $W_i$ can be computed as:

3 $$\begin{aligned} rs_{i,j} = \sum \limits _{k=1}^{n_i} w_{i,j,k} \end{aligned}$$

where $rs_{i,j}$ represents the sum of the *j*th row of $W_i$.

The transition matrix $T_i$ can be computed by dividing each element of $W_i$ by the corresponding row sum (Fig. 3B):

4 $$\begin{aligned} t_{i,j} = \frac{w_{i,j}}{rs_{i,j}} \end{aligned}$$

where $t_{i,j}$ represents the element of the transition matrix *T* corresponding to the TP from node *i* to node *j* in graph $G_i$. In other words, $t_{i,j}$ is the probability of node *j* being reached in one step by a random walker located in node *i*.

#### Alternative transition probability matrices

Further, two alternative transition matrices $T^a$ & $T^b$ are computed to address the problem of identical RAS values of the 0-, a-, b-, and c-series of the ganglioside metabolism pathway.

In case of $T^a$, the transition matrix is computed as described above. If $i = 1$ in $t_{i,j}$, we set $t_{i,j}$ to the TP of the incoming edge of node *i* with the largest probability. If the TP of the incoming edge used to set $t_{i,j}$ is also 1, we recursively repeat the process to find the TP of the previous edge until a value $\ne 1$ is found. If no incoming edges exist, the TP stays 1 (Fig. 3C).

In case of $T^b$, let *x* be a node in $G_i$. We can compute the transition matrix $T^b$ for $G_i$ by computing the sum of TPs along all simple paths from *x* to all other nodes in $G_i$. Specifically, for each simple path $P_{x,j}$ from *x* to *j* in $G_i$, we compute the sum of TPs along the path as follows:

5 $$\begin{aligned} t_{P_{x,j}} = \prod \limits _{(i,k) \in P_{x,j}} t_{i,k} \end{aligned}$$

where (*i*, *k*) represents the edge in $G_i$ from node *i* to node *k*. This is similar to the product rule for probability along a branch in a tree diagram. This method is illustrated in Fig. 3D.

We set $t_{P_{x,j}}$ as the new TP value of the last edge of $P_{x,j}$. As *G* is not strongly connected, we account for nodes that are not reachable from *x* by setting the TP of the respective edges to 0.

In this study, we selected lactosylceramide (C01290) as the *x*-node, since the sphingolipid metabolism pathway splits into the lacto-/neolacto-, globo-, and ganglio series at this point.

#### Alternative transition probability matrices

Finally, the original RAS matrix *W* is adjusted by *T*, $T^a$, and $T^b$, as follows, resulting in three additional weight matrices per graph $G_i$.

6 $$\begin{aligned} W_{1} = W * T \end{aligned}$$

7 $$\begin{aligned} W_{2} = W * T^a \end{aligned}$$

8 $$\begin{aligned} W_{3} = W * T^b \end{aligned}$$

Consequently, we obtain one matrix $M_{W}$, that represents unadjusted RAS values; three matrices $M_{W_{1}}$ (Eq. 6), $M_{W_{2}}$ (Eq. 7), $M_{W_{3}}$ (Eq. 8), which combine the RAS matrix with the TPs lowering the RAS values proportionally. These matrices represent $reactions \times samples$, where the value $M_{i,j}$ represents the edge weight of reaction *i* in sample *j*.

### UMAP plot generation from the adjusted RAS matrices

We generated multiple Uniform Manifold Approximation and Projection (UMAP) [47] plots using the adjusted RAS matrices as input and varied the number of neighbors. The UMAP algorithm is a nonlinear dimensionality reduction method that maps high-dimensional data onto a low-dimensional space while maintaining the local structure of the data. We visually inspected the UMAP plots to identify patterns in the data. The UMAP R package (version 0.2.10) was used. The choice of parameters in UMAP is crucial for achieving a meaningful and balanced representation of the data. UMAP has two primary parameters: *n_neighbors *and *min_dist*, which are instrumental in controlling the trade-off between local and global structure in the resulting projection. We adopted an iterative approach, investigating different *n_neighbors *and *min_dist *values for each RAS adjustment method within our study. Through this process, we aimed to find a balanced representation between the local and global structures of the data. For the GN/NB dataset the final *n_neighbors *and *min_dist *values were 3 and 0.1 across all RAS matrices. For the TARGET NB/GNB dataset we selected the value 20 for *n_neighbors *and 0.1 for *min_dist *in case of the RAS matrix and the value 12 for *n_neighbors *and 0.1 for *min_dist *for the adjusted RAS matrices. Additionally, to ensure the reproducibility of our research given UMAP’s stochastic nature, we used the *random_state *parameter to set a seed for random number generation.

### Clustering the UMAP results

The HDBSCAN clustering method of the dbscan R package (version 1.1-11) [48] was used to process the UMAP manifolds after creating the UMAP graphs. To find clusters, HDBSCAN examines local density peaks in the data. High-dimensional data with a variety of densities and shapes may be processed with this scalable technology. We adjusted the minimum size of clusters (*minPts*) by inspecting the sample number per cluster for different values of *minPts* (Fig. S8) and selected a *minPts* value, where the number of outlier samples (cluster 0) is minimal and the number of samples per cluster is moderate. For the TARGET NB/GNB dataset the *minPts*-value was set to 10 and for the GN/NB dataest the *minPts*-value was set to 5. Note, that an optimal *minPts* value strongly depends on the UMAP layout.

### Estimating the stability of UMAP results

As UMAP representations are the result of a heuristic algorithm, each time the UMAP plot is generated the position of the samples in the UMAP plot differs, although the local structure of the data is still preserved. To estimate the stability of the UMAPs and identify the number of clusters for more than one UMAP without cherry-picking one UMAP plot, we iteratively computed 1000 UMAPs on $M_{W}, \dots , M_{W_{3}}$, summed up the x- and y-coordinates of each sample, and plotted 50 random selected iterations as a line plot. Additionally, we computed the C-index, the Calinski-Harabasz index, and the silhouette score across the iterations to assess the clustering performance by using the R package clusterCrit (version 1.3.0).

### Marker identification

Marker reactions were identified for each cluster based on the results of the clustering analysis. In our study, we used the R function findMarkers from the scran package (version 1.22.1) [49] to identify markers for each cluster identified by HDBSCAN clustering. We set the pval.type to “all” and the test.type to “t” to perform a two-sample t-test between each cluster and the rest of the samples. The function returns a list of significant markers for each cluster along with their log-fold change, false-discovery rate, and *p*-values. The resulting list of markers allowed us to gain insights into the biological processes and reactions that are enriched in each cluster.

### Differential reaction activity analysis between two groups

Using the respective groups of the datasets, namely (1) NB MYCN+ against NB MYCN-, (2) GNB against NB, and (3) GN against NB, the log2 fold-change of the average adjusted RAS per reaction was calculated between the groups. The nonparametric statistical KS test can be used to compare a sample distribution to a reference or theoretical distribution. The null hypothesis is that the sample comes from the reference distribution. For each reaction the KS test was performed with a default *p*-value threshold equal to 0.05, to test if there is a significant difference in the adjusted RAS distributions across the samples in the two groups. The *p*-values were adjusted for multiple testing with the Benjamini-Hochberg (BH) method.

### Differential gene expression analysis & gene set enrichment analysis

Differential gene expression (DGE) analysis of the RNA-seq data between 19 TARGET GNB/NB samples related to the identified “cluster 1” and 136 samples of the other clusters was performed using the DESeq2 package (version 1.34.0) by fitting the negative binomial generalized linear model for each gene and using the Wald test for significance testing. Genes of the count matrix with less than 10 counts in the sum of all samples were excluded. The False Discovery Rate (FDR) was set to 0.05. Benjamini-Hochberg correction was used to obtain adjusted *p*-values. The “apeglm” log2 fold shrinkage method was used [50]. Additional file 3 provides the result of DGE.

Differentially expressed genes were used to perform enrichment analysis by testing the over-representation of Gene Ontology (GO) terms (only terms assigned to the category Biological Process were analyzed). The analysis was conducted by the R packages topGO (version 2.46.0) [51] and pcaExplorer (version 2.20.2) [52]. The elim algorithm [53] was used for the enrichment testing as well as Fisher’s exact test. This step was performed independently for up- and down-regulated genes. All expressed genes of the RNA-seq data were used as background genes. An enrichment map was computed by the R package GeneTonic [54].

## Results

### Comparison of RAS Adjustment Methods: Investigating Differential Reaction Activities of the GSL pathway using the TARGET GNB/NB dataset

**Fig. 4:**
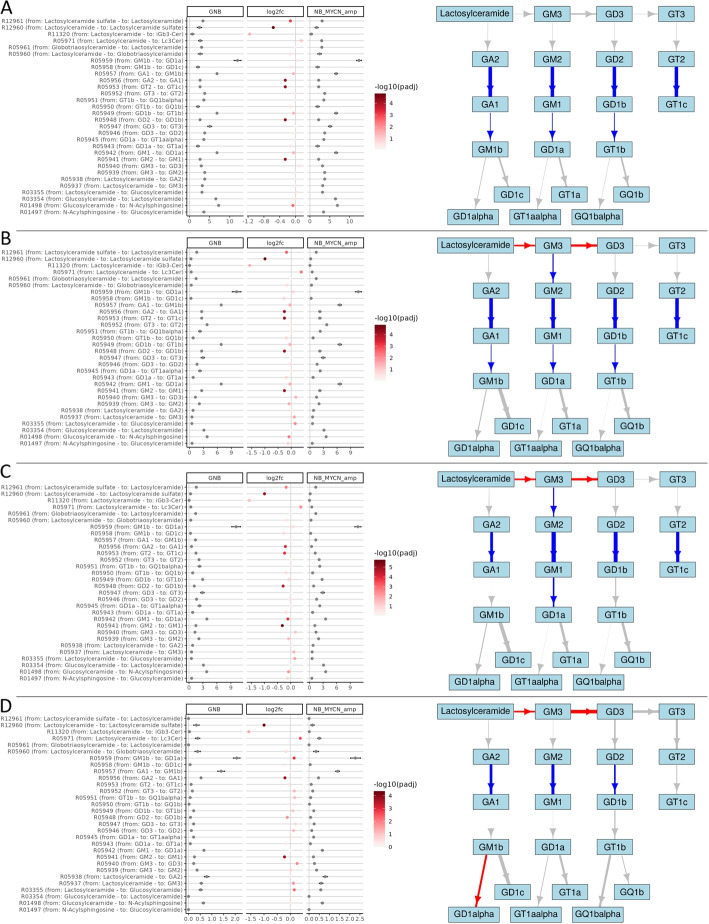
Comparison of RAS-adjusted methods on the GSL pathway between NB +MYCN and GNB. Left dot plots illustrate the adjusted RAS values of NB +MYCN (“NB_MYCN_amp” in figure) and GNB, as well as the log2 fold-change between the two groups. The whiskers indicate the standard deviation. The color intensity of log2 fold-change points indicates the negative decimal logarithm of the adjusted *p*-values. On the right, the subgraph containing the GSL pathway is visualized. Nodes represent the gangliosides and edges are the metabolic reactions (arrows). The arrow color describes the log2 fold-change direction, where red means that the reaction is more active in NB +MYCN compared to GNB and blue means the reaction is stronger in GNB compared to NB +MYCN. Gray arrows represent reactions that are not significant (adjusted *p*-value $> 0.05$). The thickness of the arrow represents the relative log2 fold-change. The subplots show A) values from $M_{W}$ (RAS values), B) $M_{W_{1}}$ (RAS values adjusted by TP), C) $M_{W_{2}}$ (RAS adjusted by the recursive TP), and D) $M_{W_{3}}$ (RAS adjusted by TP of paths)

**Fig. 5:**
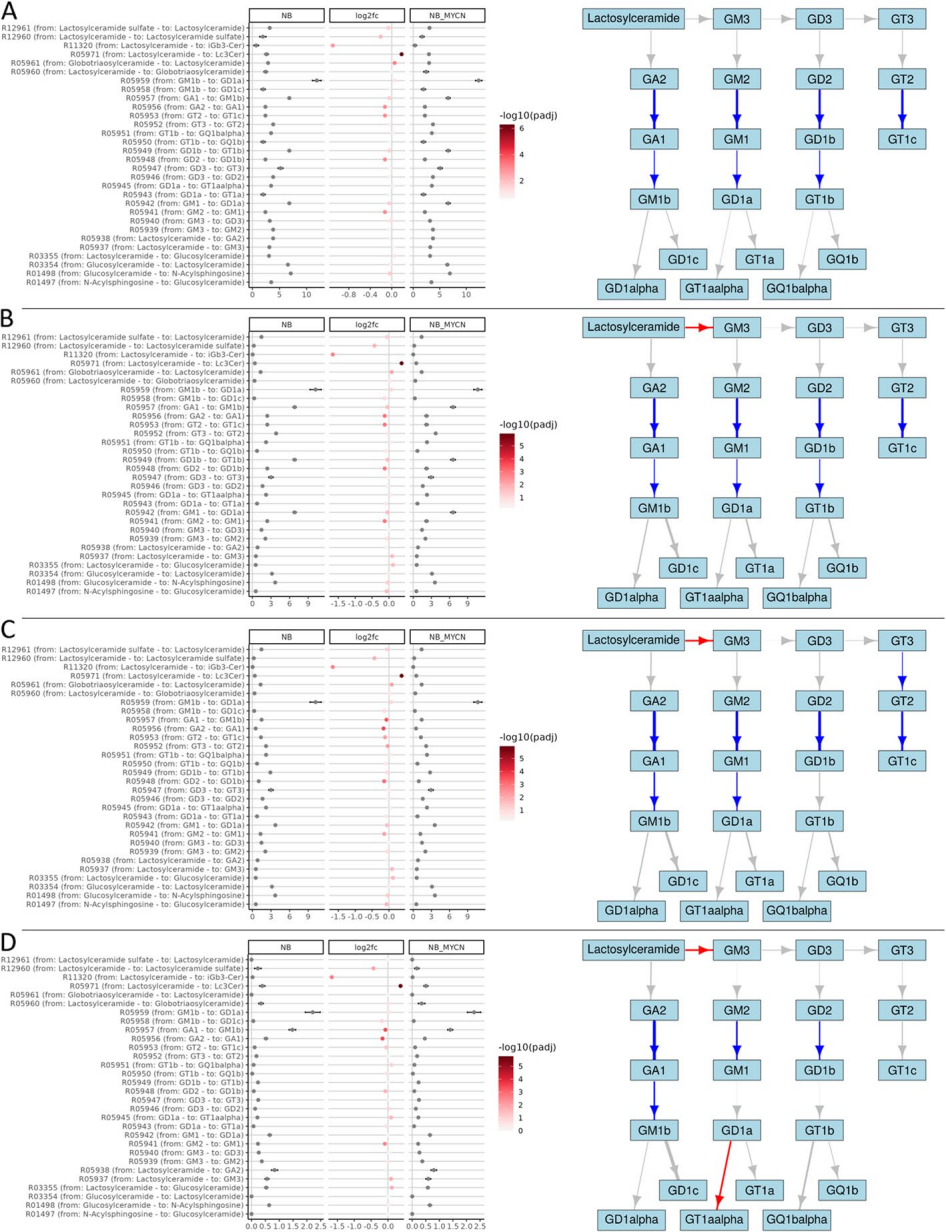
Comparison of RAS-adjusted methods on the GSL pathway between NB -MYCN and NB +MYCN. Left dot plots illustrate the adjusted RAS values of NB -MYCN (NB) and NB +MYCN (NB_MYCN), as well as the log2 fold-change between the two groups. The whiskers indicate the standard deviation. The color intensity of log2 fold-change points indicates the negative decimal logarithm of the adjusted *p*-values. On the right, the subgraph containing the GSL pathway is visualized. Nodes represent the gangliosides and edges are the metabolic reactions (arrows). The arrow color describes the log2 fold-change direction, where red means that the reaction is more active in NB +MYCN compared to NB -MYCN and blue means the reaction is stronger in NB -MYCN compared to NB +MYCN. Gray arrows represent reactions that are not significant (adjusted *p*-value $> 0.05$). The thickness of the arrow represents the relative log2 fold-change. The subplots show A) values from $M_{W}$ (RAS values), B) $M_{W_{1}}$ (RAS values adjusted by TP), C) $M_{W_{2}}$ (RAS adjusted by the recursive TP), and D) $M_{W_{3}}$ (RAS adjusted by TP of paths)

In this study, we utilized the two datasets, TARGET GNB/NB and the GN/NB dataset, to investigate the transcriptional regulation of the ganglioside metabolism pathway in NB and GNB or NB and GN, with a particular focus on distinguishing the individual series involved in ganglioside metabolism. In the following, the RAS values of the TARGET NB/GNB dataset were calculated based on the final metabolic graph and the respective gene expression values of the samples. The RAS matrix W was adjusted with one of the three transition probability matrices described in the “Methods” section, resulting in the four matrices $M_{W}$, $M_{W_{1}}$, $M_{W_{2}}$, $M_{W_{3}}$, each of which consists of reactions $\times$ samples, and where the value $M_{i,j}$ denotes the edge weight of reaction *i* in sample *j*. We analyzed three comparisons based on the TARGET GNB/NB dataset, 1) NB +MYCN vs. GNB (Fig. 4), 2) NB +MYCN vs. NB -MYCN (Fig. 5), and 3) NB -MYCN vs. GNB (Fig. S2). A similar comparison was performed on the GN/NB dataset and can be found in Fig. S3. Log2 fold-changes and RAS values for all comparisons are provided in Additional file 4.

Figure 4 illustrates the first comparison (NB +MYCN vs. GNB). A, B, C, and D represent the four matrices. The figure is reduced to the reactions relevant to ganglioside biosynthesis. The absolute values of the respective comparison group and the log2 fold-change are shown on the left. The coloring of the log2 fold-change dots reflects the negative decimal-logarithmic *p*-value. On the right side, the ganglioside pathway as part of the GSL metabolic graph is shown. Non-significant reactions (with an adjusted *p*-value $> 0.05$) are grayed. Reactions that are more active in NB +MYCN are red and those that are more active in GNB are blue. The width of the arrows indicates the relative log2 fold-change. As expected, in Fig. 4A the log2 fold-changes as well as the absolute values are the same for reactions with the same enzymes. One can only see the tendency that GNBs tend to form complex gangliosides. While this tendency changes and becomes more fine-grained when looking at the adjusted RAS values. Over the plots of Fig. 4 that show adjusted RAS (Fig. 4B, C, and D), the reaction R05940 (from GM3 to GD2, involving *ST8SIA1*) remains significant and indicates a higher activity in NB +MYCN. The reaction R05937 (from lactosylceramide to GM3, involving *ST3GAL5*) is also significant and higher in NB +MYCN but indicates a smaller log2 fold change and a larger *p*-value. Another notable reaction that is significantly higher in NB +MYCN and shows a big fold-change, is R05971 (from lactosylceramide to Lc3Cer, involving *B3GNT5*) suggesting a tendency of NB +MYCN to enter the lacto- and neolacto series. Also as expected, in Fig. 4B only the first reaction values of the four series are changed. Figure 4D indicates that the reaction from GM1b to GD1alpha of the 0-series is significantly more active in NB +MYCN.

Overall, the scores between NB +MYCN and GNB are not highly differentially expressed and relatively similar. Only few reactions differ more than 10% between the two entities (not shown in the figure), indicating fine-grained genomic changes that result in relatively small log2 fold-changes. In summary, depending on the adjusted method, different ganglioside series are predicted to be active in NB +MYCN and GNB. Particularly, the adjustment methods that discriminate all reactions between the four series (Fig. 4C and D) suggest the a-series to be more active in GNB, while the higher activity of the reactions R05937 & R05940 in NB +MYCN hints to an accumulation of gangliosides of the b-, or c- series.

Similar results can be observed in the comparison between NB and GN (Fig. S3). Although more reactions are significant due to the low sample number, there seems to be a similar tendency of GN to metabolize the simpler gangliosides to more complex ones, because the reactions R05941 & R05948 show a larger log2 fold-change compared to NB (e.g. Fig. S3B). Reaction R05940 is also similar to the above comparison of GNB and NB hinting towards a higher activity of the b-series. However, there are also differences, so the large fold-change values of reactions R05938 and R05947 can be interpreted as an increased activity of the 0- and c-series in NB.

In contrast to the previous comparison, the differential reaction activity between NB -MYCN and NB +MYCN is overall smaller, since it is the same tumor entity. Notably, the small differences are also reflected in the fact that the changes between the adjustments are more subtle. In all four subfigures of Fig. 5, there is a trend for the upstream reaction responsible for the more complex gangliosides to have slightly higher median activity in NB -MYCN compared to samples with MYCN amplification. This would indicate that MYCN amplified samples tend to have a higher GD2 concentration, as GD2 is further metabolized in -MYCN samples. The patterns in Fig. 5B, C, and D indicate that reaction R05971, in which lactosylceramide is metabolized to GM3, is significantly more active in MYCN-amplified NBs. The three reactions R05957, R05949, and R05942, which show particularly high absolute adjusted RAS values in Fig. 5A and B, are reduced by the two alternative adjustment methods and brought to a similar activity level of other reactions. Whereas the absolute RAS values of reaction R05959 remain constantly high across the four subfigures. Similarly to the previous comparison, the reaction R05971 indicates a large positive fold-change and is highly significant. Again, this could mean a stronger tendency of NB +MYCN to enter the lacto- and neolacto series. Overall, the scores between NB +MYCN and NB -MYCN are not highly differentially expressed and are very similar, resulting in a relatively small log2 fold-change range. These results suggest that simple gangliosides may accumulate in samples with a MYCN amplification.

The metabolic graph used in our analysis results from the integration of four KEGG pathways. We examined whether there is an NT type-specific difference in the activity of the individual pathways of the graph. The average activity of the RAS was calculated as the sum of all reactions involved in the respective pathway. As shown in Fig. S4, there are no major differences in the overall activity of each pathway between NB & GN in the respective dataset, and NB -MYCN, NB +MYCN, and GNB of the TARGET GNB/NB dataset. In both comparisons, the sphingolipid metabolism pathway in NB & NB +MYCN tends to be less active than in GN and GNB. This can be explained by strong activity differences of sulfate and sulfatide metabolic reactions.

### Correlation analysis of GD2 relevant genes and MYCN

The previous results suggest that the expression of gangliosides in NT changes depending on the subtype (NB, GNB) and the MYCN amplification status. This may affect the expression of clinically relevant gangliosides such as GD2. Therefore, we further analyzed the expression of genes required for GD2 synthesis and MYCN in the GN/NB and TARGET NB/GNB datasets.

**Fig. 6:**
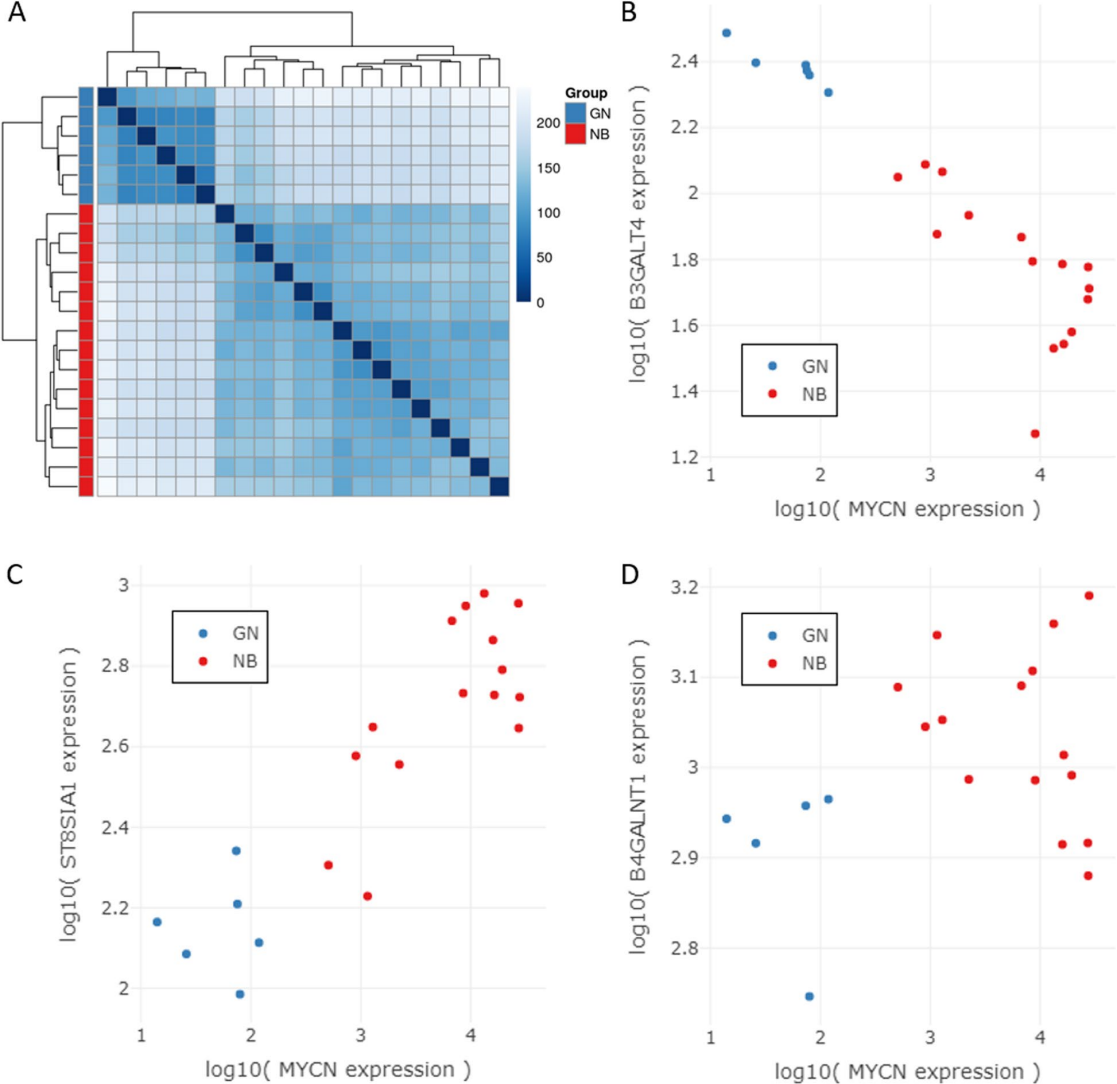
**A** Heatmap illustrating the sample-to-sample distances of variance stabilized RNA-seq data (GN/NB dataset); GN (*n* = 6), and NB (*n* = 15); Intensity of the blue color indicates high and low similarity between samples. **B** Gene expression values of *MYCN* vs. *B3GALT4*. **C** Gene expression values of *MYCN* vs. *ST8SIA1*. **D** Gene expression values of *MYCN* vs. *B4GALNT1*. Red and blue dots indicate NB and GN samples, respectively

Our analysis reveals that the biological samples of the GN/NB dataset exhibited a clustering pattern that was highly dependent on the tumor entity, as determined by hierarchical clustering (Fig. 6A). In GN, *B3GALT4* exhibited notably high expression levels, while *ST8SIA1* displayed relatively lower expression levels when compared to NB supporting our previous finding suggesting the expression of the a-series and of complex gangliosides in more differentiated tumors (Fig. S3) Moreover, we observed a strong negative correlation between the gene expression of *MYCN* and *B3GALT4* (*r* = -0.92) (Fig. 6B), as well as a strong positive correlation between *MYCN* and *ST8SIA1* (r = 0.89) (Fig. 6C). Interestingly, the gene *B4GALNT1*, which is associated with the ganglioside pathway and is involved in the biosynthesis of GD2, did not exhibit a high correlation with *MYCN* (r = 0.26) (Fig. 6D). GNs show the lowest expression of *MYCN* and the highest expression of *B3GALT4* suggesting an involvement of *MYCN* in the further metabolization of GD2 to GD1b, as expected in more differentiated tumors.

**Fig. 7:**
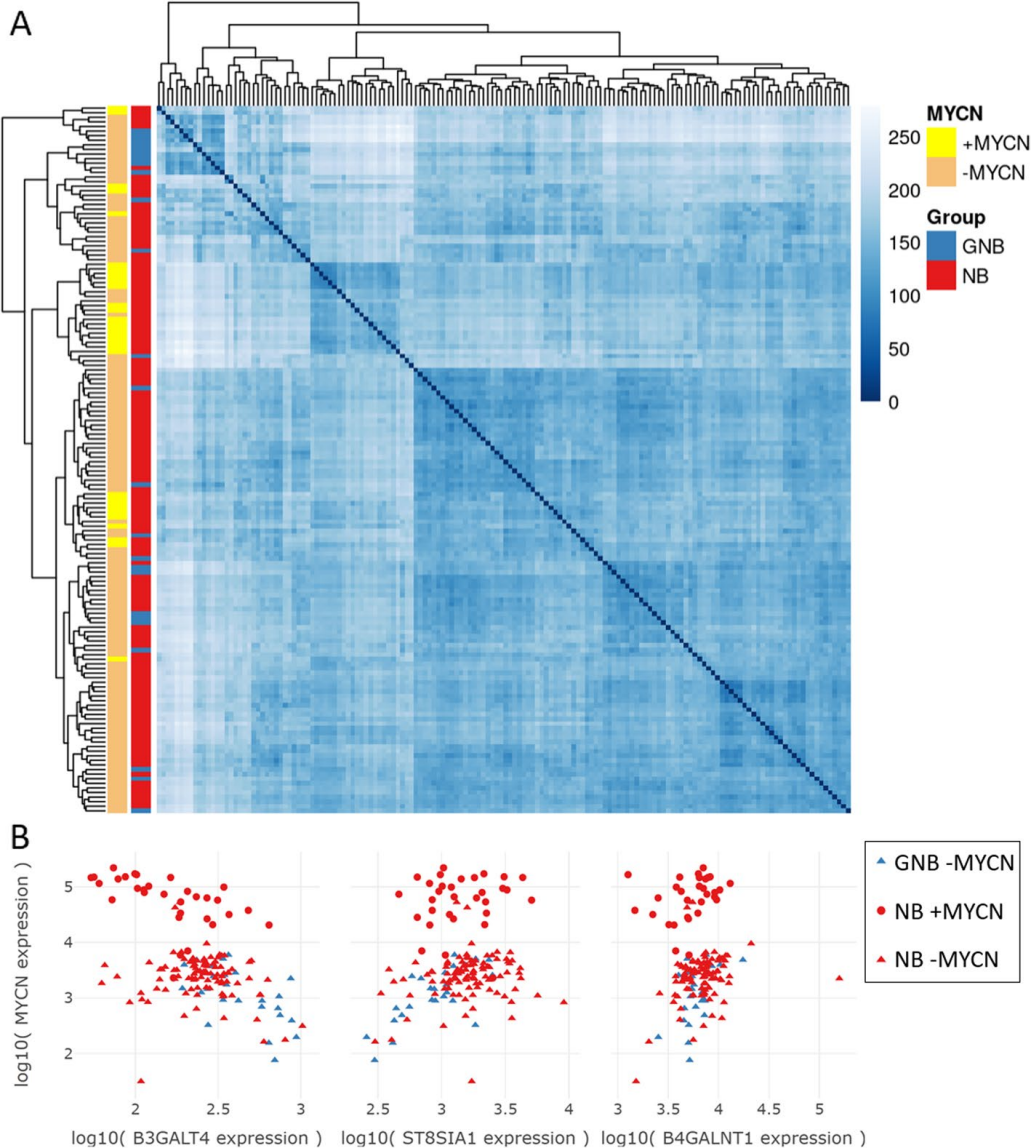
**A** Heatmap illustrating the sample-to-sample distances of variance stabilized RNA-seq data (TARGET NB/GNB) (*n* = 154); blue annotation = GNB (*n* = 25), and red annotation = NB (*n* = 129); yellow annotation = +MYCN (*n* = 31), orange annotation = -MYCN (*n* = 123); Intensity of the blue color indicate high and low similarity between samples. **B** Gene expression values of *MYCN* vs. *B3GALT4*, *ST8SIA1*, and *B4GALNT1*. Red and blue data points indicate NB and GNB samples respectively. Round-shaped data points symbolize +MYCN samples and triangle-shaped ones show -MYCN samples

In contrast, the TARGET GNB/NB dataset does not show a clear clustering of samples based on the tumor entity or the MYCN amplification status in the overall distance matrix (Fig. 7A). Nevertheless, the MYCN status seems to cluster better than the tumor entity. We assessed the Pearson correlation between *MYCN* expression and the three genes of the ganglioside metabolism related to GD2 biosynthesis within each of the NT groups (NB +MYCN, NB -MYCN, and GNB) and across different combinations of these groups to identify co-expression or co-regulation patterns, as well as to compare gene relationships between the subtypes (Table 1). The correlation coefficient r shows strong negative values between *MYCN* and *B3GALT4* within MYCN amplified NB samples, GNB, their combination, as well as across all samples. Between *MYCN* and *ST8SIA1* a strong positive correlation exists only within GNB and in combination with NB +MYCN & GNB. Although GNB samples have a strong positive correlation between *MYCN* and *B4GALNT*, the other NT subtypes and combinations of subtypes do not indicate a co-expression pattern. The visualization of the three GD2-related genes vs. *MYCN* can be seen in Fig. 7B.

Taken together, these data support the role of *MYCN* in the differentiation of NT tumors [55] by regulating the expression of the *B3GALT4* gene, which is required for the synthesis of complex gangliosides.

**Table 1:**
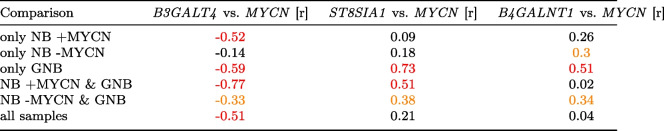
Pearson correlation coefficient between expression values of GD2-related genes and MYCN for different sample subsets

### Unsupervised data exploration of GN/NB dataset indicates distinct subgroups in NB based on adjusted RAS values

**Fig. 8:**
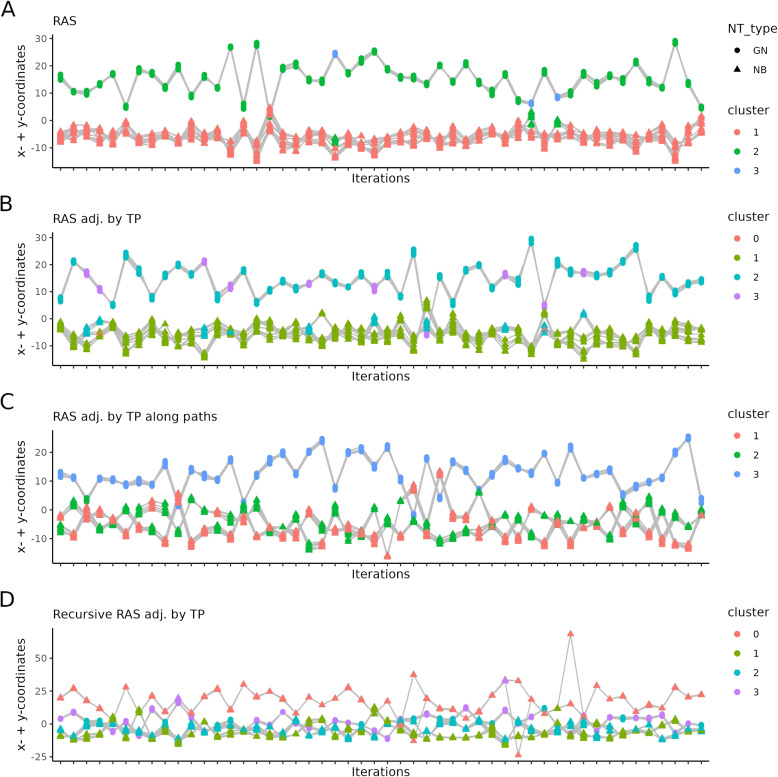
Stability assessment of the unsupervised clustering of the adjusted RAS matrices. UMAP dimensionality reduction with HDBSCAN clustering was performed on 21 samples of the GN/NB dataset over 1000 iterations. UMAP’s parameter *n_neighbor *attribute was set to 3. The subplots show 50 random selected iterations of UMAP followed by HDBSCAN clustering (x-axis) for A) $M_{W}$ (RAS values), B) $M_{W_{1}}$ (RAS values adjusted by TP), C) $M_{W_{2}}$ (RAS adjusted by the path-specific TP), and D) $M_{W_{3}}$ (RAS adjusted by the recursive TP). The color indicates the cluster of the respective sample. GN is represented through a circle, while NBs are rectangular. The same samples are connected with lines between the iterations, indicating cluster stability. The y-axis represents the sum of x- and y-coordinates of the UMAP per sample

Using an unsupervised learning approach, our goal was to show that by adjusting the RAS values of the GSL metabolism and differentiating the ganglioside series, the identification of novel subgroups would potentially be possible. Therefore, we first examined the GN/NB dataset with UMAP and performed HDBSCAN clustering on the UMAP coordinates to determine visually appropriate parameters. To estimate the stability of the identified clusters in dependence of the adjustment method, we calculated UMAPs and HDBSCAN clustering on 1000 iterations and summed the x- and y-coordinates per sample. The results were visualized as line plots per adjustment method in Fig. 8.

In Fig. 8A, it can be seen that the NT types based on RAS form two clusters over all iterations. By adjusting the RAS values with the TP, these two NT type based clusters can be further observed (Fig. 8B). By applying the third adjustment method based on TP along defined paths, two further clusters become apparent within the NB type (Fig. 8C). In this case, the number of variables for the UMAP calculation are reduced because reactions outside of possible paths are removed. As the TP differs significantly in dependence of the path length, the TP matrix was scaled to make the scores homoscedastic for the UMAP. The adjustment method based on the recursive TP identifies most of the time three clusters but shows a weaker cluster stability, as more outliers are found (HDBSCAN annotates outlier samples as a pseudo-cluster 0) and more samples are switching between the clusters (Fig. 8D).

As additional diagnostic metrics for the clustering performance, the C-index, the Calinski-Harabasz index, and the silhouette score were computed across the iterations (Fig. S9). The C-index in both datasets based on the adjusted/non-adjusted RAS values is in the low range, indicating coherent and well-separated clusters. Due to the high homogeneity of the NB/GNB dataset, the C-index is slightly higher compared to the GN/NB dataset. The clear separation in the GN/NB dataset is also evident in the high values of the silhouette score. Interestingly, the RAS adjusted by the recursive paths in the NB/GNB dataset leads to a clear formation of two widely separated clusters. The higher values of the Calinski-Harabasz index and the silhouette score support this observation. In summary, although the scatter between the iterations is higher in the more homogeneous NB/GNB dataset than in the NB/GN dataset, it can be said that the results of the iterations are consistent, except for a few outliers.

**Fig. 9:**
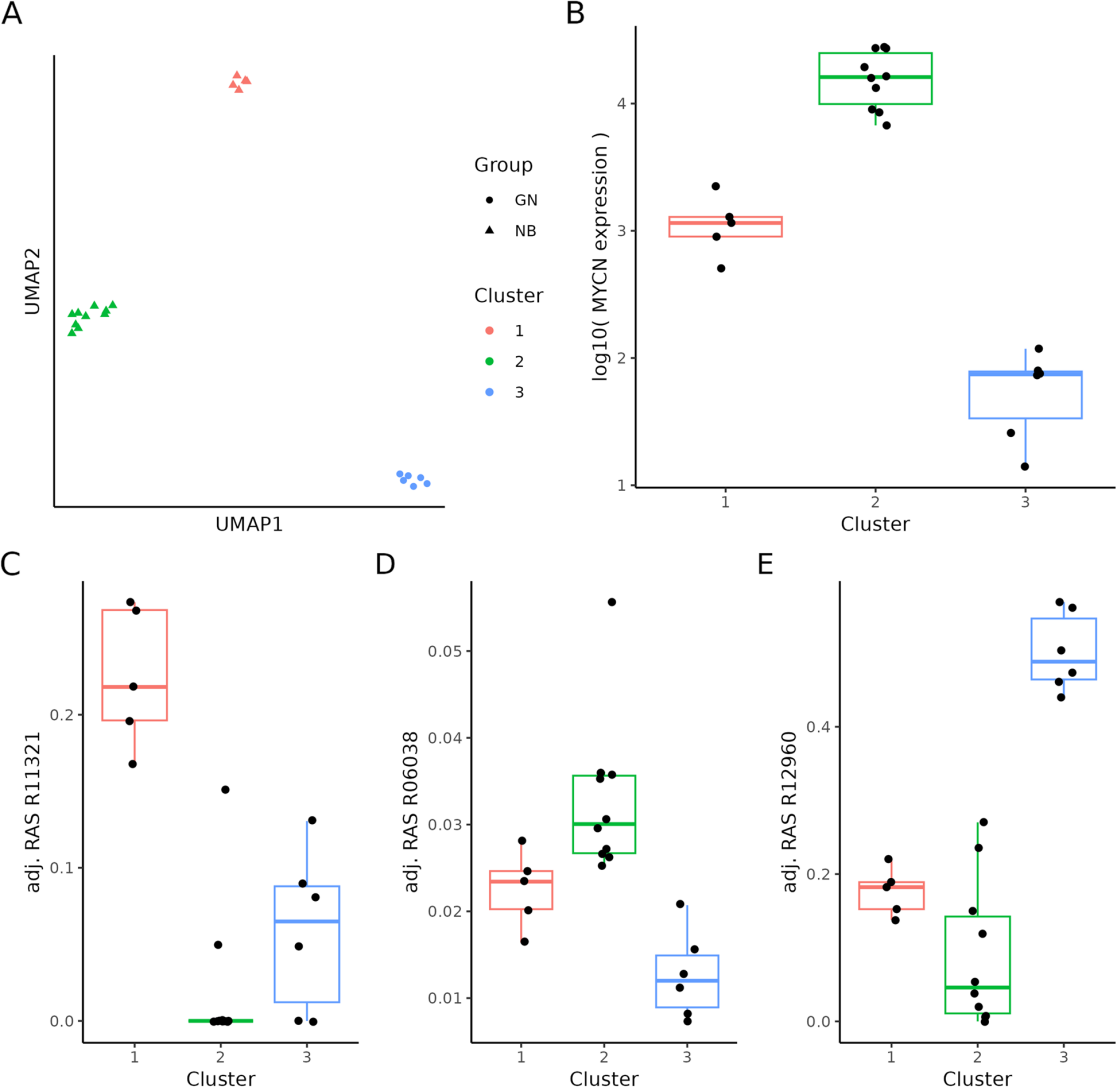
Unsupervised clustering and identification of marker reactions. **A** The HDBSCAN clustering of the UMAP coordinates based on the $M_{W_{3}}$ (RAS values adjusted by TP along paths) shows a clear separation between GN and NB, as well as a separation of NB samples into two distinct groups. Round symbols represent GN and triangles are NB samples. **B** Box plot of normalized MYCN expression grouped by identified clusters. With a t-test statistic we demonstrate characteristic reactions per cluster. As an example, the box plot in **C**) shows the adjustment RAS values of reaction R11321 characteristically for cluster 1, **D**) the reaction R06038 characteristically for cluster 2, and **E**) the reaction R12960 characteristically for cluster 3. Boxes range from first to third quantile, the middle line indicates the median, the whiskers show the highest and lowest values no further than $1.5*IQR$ from the hinge. Black dots represent the samples

Using a UMAP result calculated on the path-based TP-adjusted RAS matrix as an example (Fig. 9A), we demonstrate the further procedure to identify characteristic reactions for the respective clusters applying t-tests. For each cluster, we have chosen one exemplary reaction with the highest *p*-value for visualization. In Fig. 9C the reaction R11321, which takes part in the globo series of the GSL biosynthesis pathway and is represented by the gene *B3GALNT1*, shows a significant *p*-value of 0.0001 and FDR of 0.01 for cluster 1. The reaction R06038, which participates in the lacto- and neolacto series of the GSL biosynthesis pathway and is represented by the fucosyltransferase (FUT) genes, shows a significant *p*-value of 0.0001 and FDR of 0.003 for cluster 2 (Fig. 9D). Lastly, reaction R12960 characterizes the third cluster (GN samples), which is responsible for the metabolism of lactosylceramide sulfate by galactosylceramide sulfotransferase with a *p*-value smaller 0.0001 and FDR $< 0.0001$) (Fig. 9E). In accord to these results, the presence of sulfatides is expected to be high in Schwannian stroma, which is the main component of GN [19].

In addition, we checked the correlation between the identified clusters and the *MYCN* expression. As shown in Fig. 9B, there is indeed a difference between the *MYCN* expression of NBs from clusters 1 and 2. As already shown in Fig. 6, one can see the clear difference in *MYCN* expression between NB (clusters 1 and 2) and GN (cluster 3).

In summary, these results suggest that unsupervised data exploration techniques can be applied to the adjusted weighting schemes and yield additional findings on subtypes and their specific characteristics. Our analysis suggests the presence of two NB subclusters with an altered sphingolipid composition. One of the subclusters is characterized by, among other factors, increased activity of the FUT enzymes and a correlation with the *MYCN* gene expression.

### Unsupervised learning of TARGET GNB/NB samples indicates a Fucosyltransferase driven subcluster

**Fig. 10:**
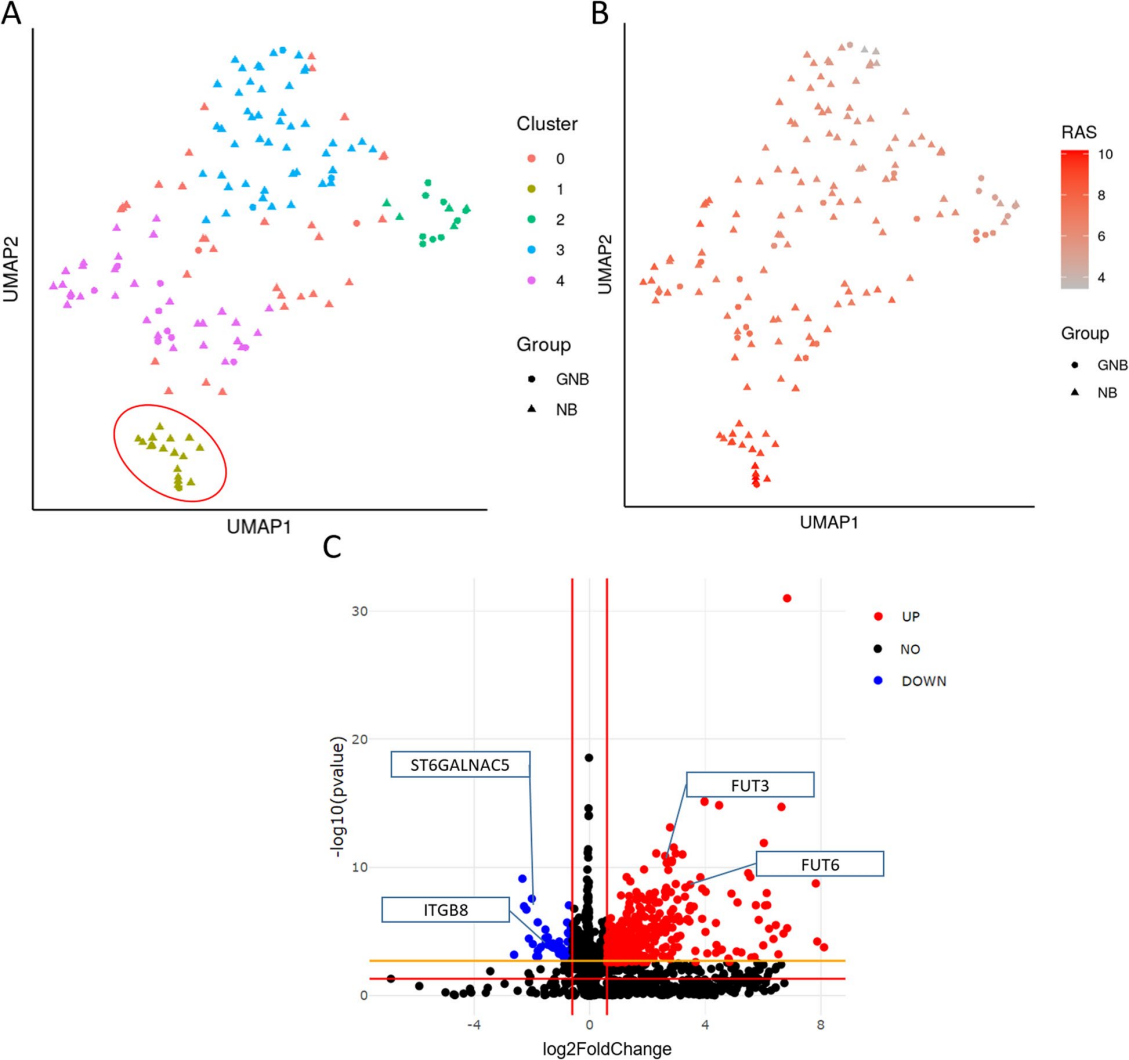
Unsupervised clustering and identification of marker reactions. **A** One representative UMAP representation out of the 1000 computed iterations, colored by the HDBSCAN clustering (in accord with the HDBSCAN method samples of cluster 0 are identified as outliers). Circles represent GNB and triangles are NB samples. The red selection indicates samples of cluster 1. **B** The same UMAP representation as in **B**) colored by the computed values for the R06025 reaction, which was identified as highly characteristically for cluster 1. **C** Volcano plot of differentially expressed genes between samples of cluster 1 and all other samples. Red dots denote upregulated genes in samples of cluster 1 compared to samples of all other clusters. Blue dots show downregulated genes in cluster 1. Not significant expressed genes are black. The red lines indicate the *p*-value threshold of 0.05, and log2 fold change thresholds of 0.6 and -0.6. The orange line indicates the adjusted *p*-value threshold of 0.05. The genes *FUT3/6*, *ITGB8*, and *ST6GALNAC5* were additionally labeled

We have also applied the unsupervised procedure described above to the TARGET GNB/NB dataset. We computed the UMAP on the unadjusted RAS values for the following analysis (Fig. 10A). Cluster 1 shows a high similarity of samples and was selected for further investigation.

The identification of the marker reactions showed that in cluster 1, among others, the reactions involving genes of fucosyltransferases (*FUT3*, *FUT4*, *FUT5*, *FUT6*, and *FUT9*), e.g. reaction R06025 of the lacto- and neolacto series, are significantly more active with $p < 0.0001$ and FDR $< 0.001$ than in the samples of the remaining clusters (Fig. 10B). Since the analysis was performed on unadjusted RAS values, all reactions involving all named FUTs have the same *p*-values and FDRs. To further investigate the transcriptional deregulation of cluster 1, differential gene expression analysis was performed between the cluster 1 samples against all remaining samples (Fig. 10C). This identified 91 downregulated and 498 upregulated genes that had an adjusted *p*-value $< 0.05$ and a log2 fold-change $< -0.6$ or $> 0.6$, which corresponds to a fold-change of 1.5 in gene expression (Additional file 3). Additionally, a heatmap of the downregulated genes can be found in Fig. S6. Among the upregulated genes, the *FUT3* and *FUT6* genes are found, as expected. Among the downregulated genes, genes of the GSL pathway are also present, namely *ITGB8* and *ST6GALNAC5*. In Fig. S5 we further investiated the association of *MYCN* expression across the UMAP representation. The specific cluster under investigation comprises only a single sample exhibiting a *MYCN* expression exceeding 4. We could not detect any discernible correlation in *MYCN* expression patterns within the UMAP. This observation suggests that the impact of *MYCN* may not be sufficiently robust to manifest discernibly in the GSL profile.

**Fig. 11:**
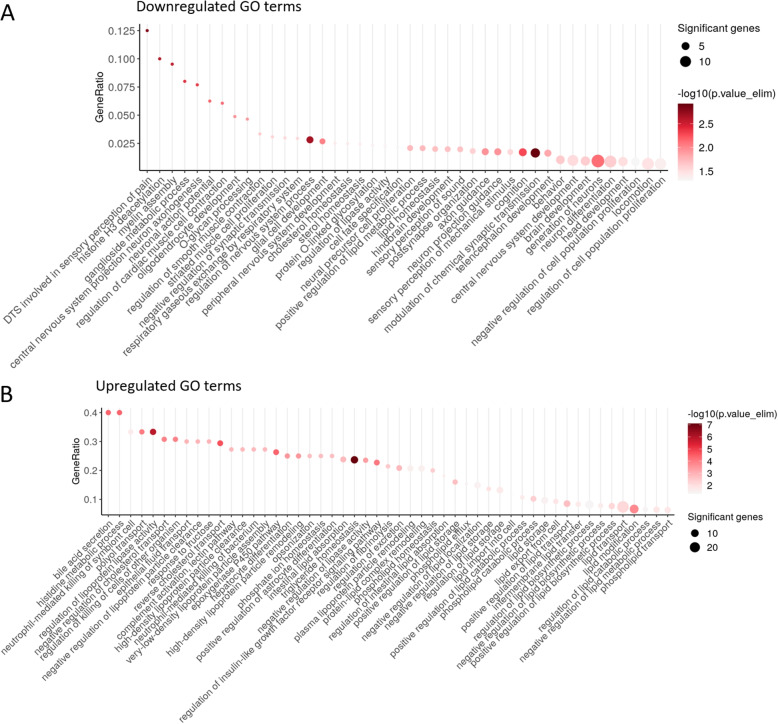
GO term enrichment analysis of up- and downregulated genes. **A** Dot plot of the GO enrichment analysis results, highlighting GO pathways enriched in the downregulated genes. Only GO terms with a *p*-value $< 0.05$ and more than one significant gene are shown. The color scale represents the negative logarithm of the *p*-value, while the dot size indicates the number of significant genes identified in each GO term. **B** Dot plot of GO pathways enriched in the upregulated genes. Due to the identification of over 500 identified GO pathways with a *p*-value $< 0.05$ and and more than one significant gene, the subset shown includes only those GO terms with a GeneRatio $> 0.2$. Additionally, several lipid-related pathways are highlighted as examples. The color scale denotes the negative logarithm of the *p*-value, and the dot size represents the number of significant genes associated with each GO term. The GeneRatio is calculated as the number of significant genes found in a given GO term divided by the total number of genes annotated to that GO term

The differentially expressed genes were used for GO term enrichment analysis in the next step. In total 92 enriched GO terms with a *p*-value (elim) $< 0.05$ were identified based on the downregulated genes and 629 enriched GO terms with a *p*-value (elim) $< 0.05$ were identified based on the upregulated genes. The downregulated genes indicated decreased activity of histone H3 deacetylation, myelin assembly, lower regulation of synaptic transmission, and glial cell development (Fig. 11A). Furthermore, the GO terms generation of neurons, neuron differentiation, brain development, positive regulation of lipid metabolic process, and lipid homeostasis are significantly enriched, yet the gene ratio between the differential genes and the term annotated to the GO term is lower (Additional file 3). In Fig. S7 we further investigated the enrichment map of the GO enriched terms from downregulated genes in cluster 1 and identified six communities. Two major communities indicate an association with 1) neuron and nervous system related terms (green), and 2) lipid-related and regulatory terms. Among GO terms enriched within upregulated genes, we detected the following pathways standing out in particular with a high gene ratio ($> 0.2$) and a particularly low *p*-value: histidine metabolic process, regulation of lipoprotein lipase activity, negative regulation of cholesterol transport, reverse cholesterol transport, epoxygenase P450 pathway, intestinal lipid absorption, and triglyceride homeostasis (Fig. 11B).

Taken together, we investigated the GSL metabolism patterns with a particular focus on the ganglioside metabolism pathway in NB, GNB, and GN using the introduced adjusted RAS values. Our analysis revealed subtle but significant differences in gene expression-driven reaction activities. Notably, comparisons between MYCN-amplified NB and GNB suggested fine-grained transcriptomic changes, indicating distinct ganglioside series activities. Correlation analyses underscored the influence of tumor entity and MYCN amplification on ganglioside expression and suggested an involvement of MYCN in the accumulation of GD2. Unsupervised techniques, such as UMAP and HDBSCAN clustering, showed an association between the clusters with the tumor type in case of the NB/GN dataset and revealed distinct subgroups within NB in both datasets. In particular, a FUT-driven subcluster within NB showed potential associations with GO terms related to neoron and nervous system, lipids, and regulatory mechanisms. The comparison between the RAS adjustment methods led to slightly different results when investigating the reaction activities of the ganglioside series and different UMAP embeddings during the unsupervised analysis, making it difficult to assess which method is more reliable. Nevertheless, the approach enabled us to better quantitatively discriminate the ganglioside series and in this context showed improvements compared to unchanged RAS values.

## Discussion

In this study, we focused on the specific issue of low substrate specificity among ganglioside metabolism enzymes and the resulting difficulty to obtain conclusions about the activity within the four series of the ganglioside metabolism when utilizing only transcriptome data. To overcome this challenge, we propose a straightforward approach that integrates network topology information and transcriptome data. We constructed a directed graph based on four GSL related metabolic pathways from the KEGG database. Transcriptome datasets of three NT entities were aggregated to RAS values for each sample and used to weighting the graph edges. Three different adjustment methods based on the TP between nodes were applied to the RAS weighing and compared among each other. This integrated methodology provides a solution for unraveling the complexities of ganglioside pathways.

Even though the study of the effect of complex metabolic processes plays an important role in many fields, such as signal transduction, biomarkers, and cancer physiology, it is still difficult to infer metabolic activity from indirect measurements, such as transcriptome data [56]. Many of the methods developed for this purpose can be divided into at least two fields, constraint-based methods (e.g., flux-based analysis) [56, 57] and graph analysis, also known as network science techniques [58–60]. In the latter case, graph construction can take different forms, such as metabolite-centric, bipartite, or reaction-centric graphs [61]. Both fields mostly use genome-scale metabolic models (GEMs), which are mathematical representations of the current state of knowledge on organ-, species-, or condition-specific metabolic properties. Constrain-based approaches, while being powerful tools, they require reaction rates, assume a steady-state condition and one or more suitable optimization objectives, which are often elusive in cancer types [32, 62, 63]. Therefore, we have intentionally refrained from using whole GEM and constraint-based methods in order to reduce the complexity of the analysis and focus only on the GSL metabolism. Acknowledging the multifaceted nature of GSL metabolism regulation, encompassing epigenetic, transcriptional, and post-translational control [64–67], we intentionally limit our model to gene expression data, as this simplification enables a focused exploration within the defined scope of our study and allows the usage of a widely available data type.

We acknowledge that a limitation of our analysis method is the involvement of many considered enzymes in reactions outside of GSL metabolism. This could obscure significant differences between the various sub-populations in our dataset. Currently, the state of the art in finding subgroups are simple RNA-Seq workflows, often in combination with other molecular data, that do not account for single reactions at all. Our primary aim was to explore the GSL metabolism and identify metabolic differences between several groups, which is not efficiently possible with the aforementioned workflows. In this context, we focused on neuroblastic tumor entities due to the existing literature on the deregulation of GSL metabolism. Given that the primary enzymes involved in ganglioside synthesis are specific to their substrates [45], we opted to simplify our approach by avoiding the use of a GEM. Regarding the technical application of the three RAS weighting methods on a GEM, we conclude that the simple TP adjustment can be applied to a whole-genome network, as it depends solely on local network topology and is unaffected by enzyme involvement in different pathways. Notably, our results from the GSL-subnetwork would remain consistent even if applied to the entire network. The recursive weighting method is also applicable to the whole metabolic graph and would not alter the results. In contrast, the path-based adjustment method relies heavily on the central node from which simple paths are calculated. Applying it to the whole network is challenging due to the absence of a central metabolic node. A potential solution could be to select a central node per pathway and prevent overlapping paths, but this approach would require further testing.

It can be observed that both adjustment methods (path-based and recursive weighting) have introduced differences in the weighting of the ganglioside series compared to the unadjusted RAS weighting. Even the simple addition of the TP with the RAS values does not provide complete differentiation. The interpretation of the results in relation to the unsupervised analysis is difficult, as the different weightings lead to different clusters. In the case of recursive adjustment, even the association of the clusters with the tumor entities dissolves and shows more outlier samples. However, this may also be due to the clustering parameters. In the comparison between two specific conditions, the differences introduced between the ganglioside series are of a subtle nature. In any case, we understand that further work is needed to evaluate the adjustment methods on other tissue and tumor entities, that may indicate stronger differences in the ganglioside patterns and to validate the results in the context of laboratory measurements, e.g. immunohistochemistry or mass spectrometry. In this first approach, we wanted to focus on well-studied neuroblastic tumor entities that have clinical relevance in terms of ganglioside metabolism.

Using this approach, we were able to predict an activation of the a-series in GNB vs. NB. This is in accord with a previous analysis showing that GNB expresses GM1a and GD1a [15]. NB samples were shown to express complex gangliosides of the b-series (GD1b and GT1b), while the presence of both the a- and b-series was observed particularly in GNB. This could be regulated by the *ST8SIA1* gene product (R05940) which is indeed more expressed in NB according to our result and underscores the hypothesis of N. W. Mabe et al. that *ST8SIA1* acts as a bottleneck on the GD2-production [26]. A lower expression of *ST8SIA1* could be a signal to the cells to enter the a-series. The co-expression of complex gangliosides of the a- and b-series is typical of mature neuronal tissues, which express very low amount of GD2, and the significantly higher expression of *B3GALT4* and lower expression of *ST8SIA1* in GN support our hypothesis that the combination of different genes is required to predict the final composition of NT. Importantly, reduced expression of *ST8SIA1* could change the composition of gangliosides towards the a-series with reduction in GD2 expression and indeed downregulation of *ST8SIA1* in NBL confers resistance to the anti-GD2 antibody [26, 68].

Our data suggest also that MYCN amplified tumors, known for their unfavorable prognosis, accumulate less complex gangliosides, driven by the activities of reactions R05956, R05941, R05948, and R05953 (all attributed to the gene *B3GALT4*). This aligns with previous data showing the absence of complex gangliosides in NB cases featuring MYCN amplification [15]. Notably, the simple ganglioside GD2 is particularly abundant in these tumors [12]. Loss of complex gangliosides has been linked to a poor prognosis in NB [11, 69]. As MYCN amplification is also associated with the differentiation of NT tumors, more complex gangliosides can be observed in GN and GNB, which are more differentiated tumor entities. This highlights the need for further investigations to unravel the relationship between MYCN amplification, regulation of differentiation, the depletion of complex gangliosides, and the concurrent accumulation of GD2.

GSL profiles are important not only in NT. GSL deregulation has been described in different tumor entities, including hard to treat tumors such as diffuse midline glioma, ependymoma [70], and triple negative breast cancer [71]. Deregulation of GSL affects the biology of the tumors. For example, expression of GD1a facilitates the adhesion of tumor cells to the endothelium and high GD2 expression has been described in metastasis of different tumor types [72, 73]. GSL can be exploited for therapy and classification. Tumors with deregulated gangliosides synthesis can be responsive to treatment with UGCG inhibitors, such miglustat and eliglustat, which are already used in the clinic for the treatment of children with Gaucher disease [15, 70, 74]. Tumors with expression of GD2 can be targeted with monoclonal antibodies [75] or CAR-T cells [76]. Finally, GSL profiles allow identifying subgroups in heterogeneous tumor entities such as medulloblastoma [15]. Therefore, the approach we propose may facilitate in the future the identification of therapeutic or diagnostic relevant GSL profiles across tumor entities.

Current prognostic markers are based on genomic alterations, particularly MYCN amplification and ALK alterations in NB. Ganglioside profiles have not been integrated into a clinical routine so far, also because of the technical requirements necessary for their quantification. The use of RNA-seq data to predict ganglioside profiles may help in the future to improve the prognosis and possibly the therapy of NT patients. Current biomarkers are indeed not sufficient to identify all patients at risk. For example, MYCN amplification is associated with a poor prognosis but children with tumors without MYCN amplification can also have a poor prognosis. GN is generally considered a benign tumor and therefore can be treated by surgery alone. However, ganglioneuroblastoma intermixed can have aggressive behavior and can need multimodal therapy. Thus, ganglioside profiling with our method may help in the future for better risk stratification of NT tumors and could help to identify subgroups with specific biological features. For instance, we were able to define a subcluster involving genes of fucosyltransferases. FUT genes are required for the synthesis of Sialyl Lewis x (SSEA1; CD15) which belongs to the neolacto series. SSEA1 is expressed in neuronal stem cells and could define a subgroup with higher potential for proliferation and self-renewal as already shown in other tumor entities [77]. Further, Cuello et al. identified an overexpression of FUT genes in MYCN amplified cell lines and patient tumors with aberrant glycosylation and agressive behaviour [78].

## Conclusions

In this paper, we described a methodological approach introducing three TP based adjustments for a RAS weighted metabolic graph with nodes as metabolites and edges representing metabolic reactions. We illustrated how these weighting schemes can be applied to a conditional comparison and an unsupervised data exploration. Furthermore, we tested our approach on three NT entities with the focus on the GSL metabolism and particularly the ganglioside biosynthesis pathway.

Our results suggest that indeed the weighting schemes make the individual series of ganglioside metabolism distinguishable, allowing a differentiated analysis of the GSL profile in NT entities. We showed that the results have a biological rationale and are largely consistent with published data on the ganglioside composition of NT. Our method may be helpful in the comparison of GSL deregulation between two specific sample groups, as well as for the identification of previously unknown cancer subgroups that exhibit distinguishability based on their sphingolipid profile. Our approach can also be used for other metabolic pathways involving enzymes that have low specificity to the substrate and participate in several metabolic reactions unlocking the potential to leverage widely available transcriptome datasets to obtain deeper insights in such clinically relevant scenarios.

## Supplementary information

Additional file 1

Additional file 2. Annotation of reactions: 1) Table with edge reaction ID, incoming edges, outgoing edges, involved genes as gene symbol and ensemble; 2) Table of considered genes and their associated KEGG pathways

Additional file 3. DGE & GO enrichment results

Additional file 4. Table containing the log2 fold-changes, p-values, adjusted p-values, and absolute values for the following comparisons across all reactions of the whole graph

## Data Availability

All analyses included in this manuscript are documented in the github repository https://github.com/arsenij-ust/NT_GSL_analysis. The GN/NB dataset analyzed during the current study are available in the Gene Expression Omnibus repository (https://www.ncbi.nlm.nih.gov/geo/) under the GEO accession GSM4437037. The TARGET GNB/NB dataset analyzed during the current study are available in the UCSC Xena database (http://xena.ucsc.edu/) under the combined cohort of TCGA, TARGET and GTEx (https://xenabrowser.net/datapages/?cohort=TCGA%20TARGET%20GTEx).

## Abbreviations

BH: Benjamini-Hochberg
DGE: Differential gene expression
FDR: False Discovery Rate
FUT: Fucosyltransferase
GEM: Genome scale metabolic model
GlcCer: Glucosyceramide
GN: Ganglioneuroma
GNB: Ganglioneuroblastoma
GO: Gene Ontology
GPR: Gene-protein-reaction
GSL: Glycosphingolipids
KEGG: Kyoto Encyclopedia of Genes and Genomes
KS: Kolmogorov-Smirnov
LacCer: Lactosylceramide
NB: Neuroblastoma
NT: Neuroblastic tumor
RAS: Reaction activity score
RNA-seq: RNA sequencing
TARGET: Therapeutically Applicable Research to Generate Effective Treatments
TP: Transition probability
UMAP: Uniform Manifold Approximation and Projection
+MYCN: MYCN-amplified samples
-MYCN: Samples without MYCN amplification
