# Supplementary material for: Unraveling the glycosphingolipid metabolism by leveraging transcriptome-weighted network analysis on neuroblastic tumors
Source: Cancer Metab. 2024 Oct 24;12:29. doi: 10.1186/s40170-024-00358-y (PMC11515559; doi:10.1186/s40170-024-00358-y)
Supplement: Supplementary file 1 — Additional file 1 [file 40170_2024_358_MOESM1_ESM.pdf]

1 Supplementary information

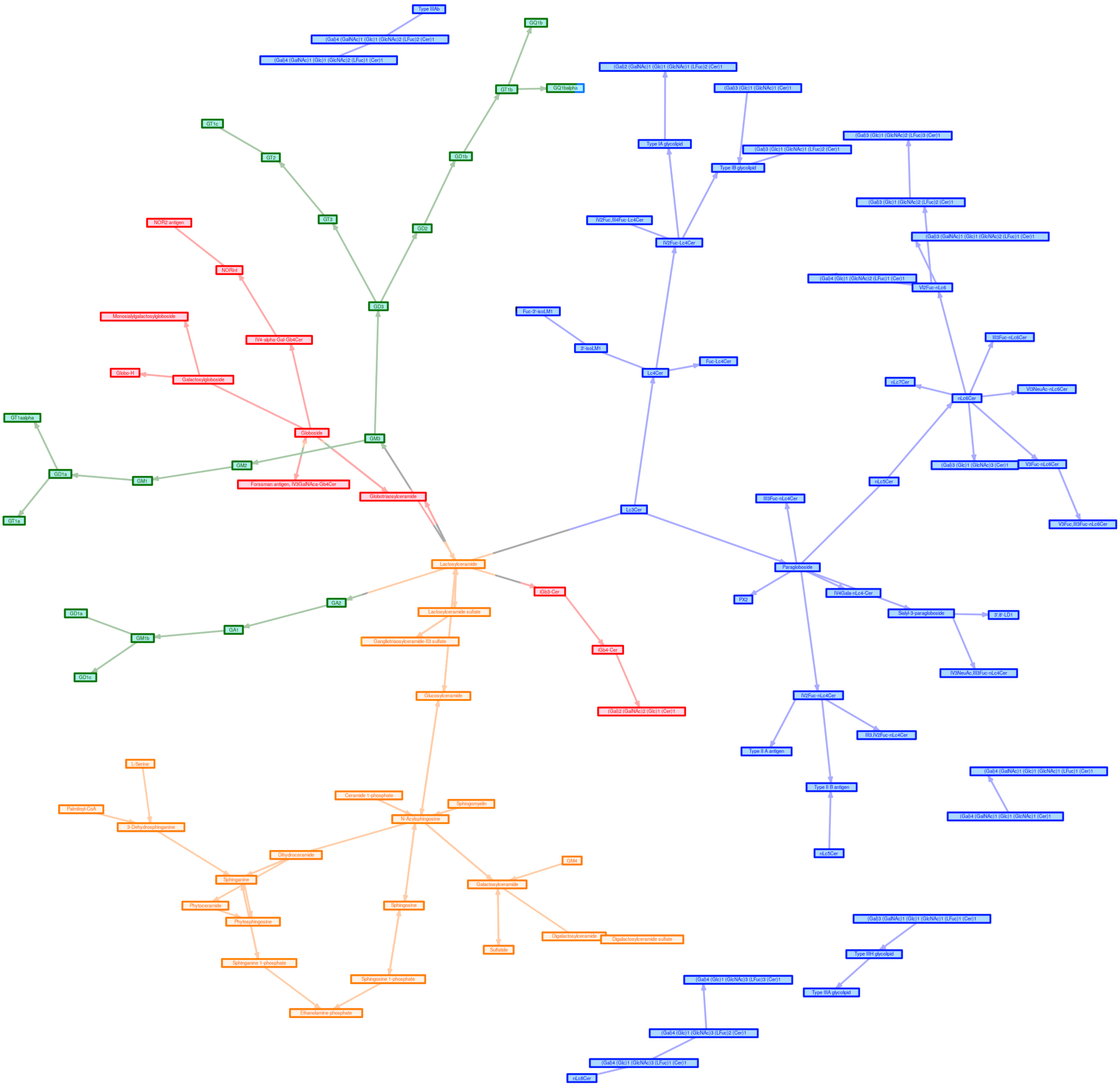

Fig. S1 Final graph containing the following four KEGG pathways. Orange: Sphingolipid metabolism pathway; Green: Glycosphingolipid biosynthesis - ganglio series; Red: Glycosphingolipid biosynthesis - globo series; Blue: Glycosphingolipid biosynthesis - lacto and neolacto series.

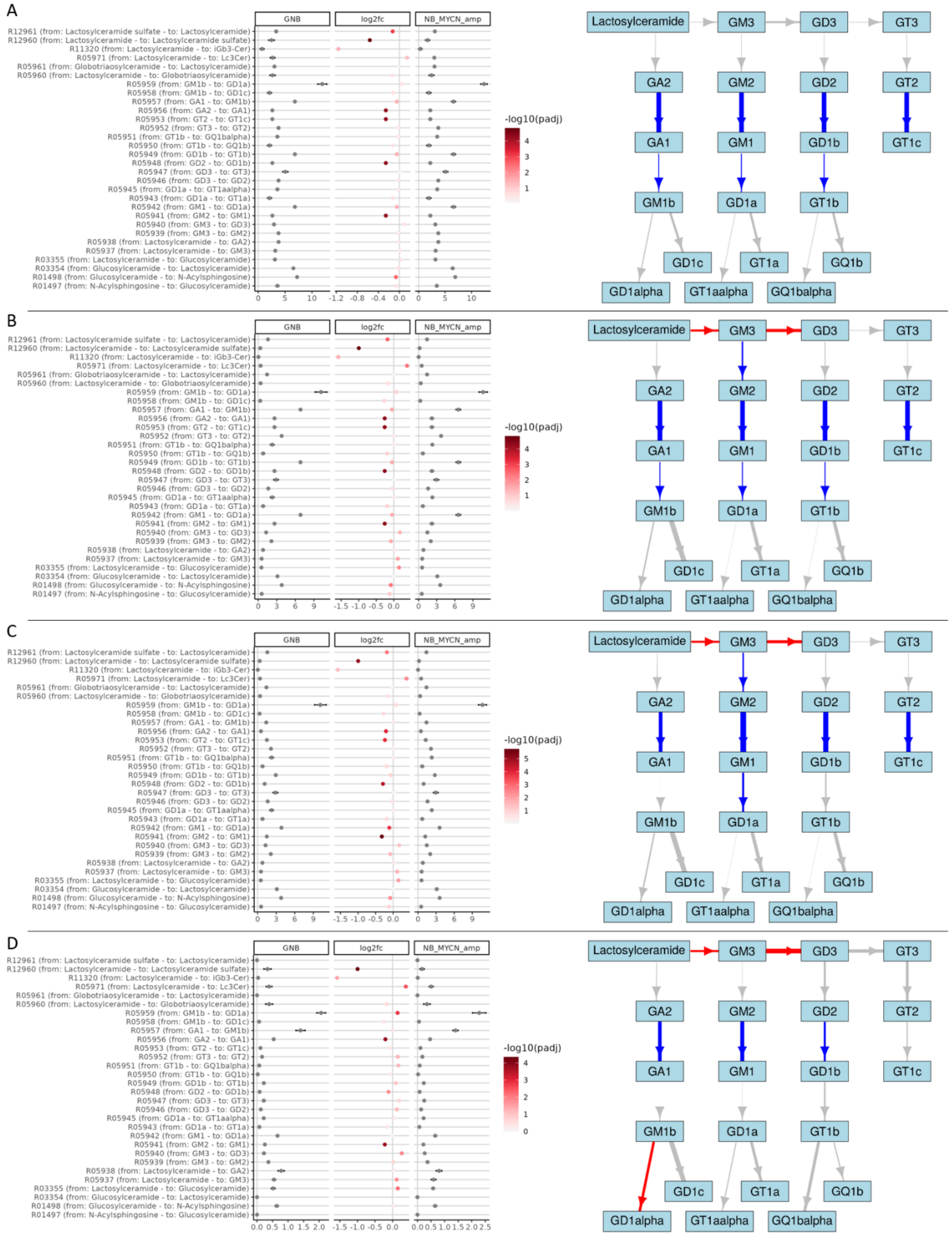

Fig. S2 Comparison of RAS-adjusted methods on the GSL pathway between NB -MYCN and GNB. Left dot plots illustrate the adjusted RAS values of NB -MYCN ("NB" in figure) and GNB, as well as the log2 fold-change between the two groups. The whiskers indicate the standard deviation. The color intensity of log2 fold-change points indicates the negative decimal logarithm of the adjusted p-values. On the right, the subgraph containing the GSL pathway is visualized. Nodes represent the gangliosides and edges are the metabolic reactions (arrows). The arrow color describes the log2 fold-change direction, where red means that the reaction is more active in NB -MYCN compared to GNB and blue means the

reaction is stronger in GNB compared to NB. Gray arrows represent reactions that are not significant (adjusted p-value > 0.05). The thickness of the arrow represents the relative log2 fold-change. The subplots show A) values from M_W (RAS values), B) M_{W1} (RAS values adjusted by TP), C) M_{W2} (RAS adjusted by the recursive TP), and D) M_{W3} (RAS adjusted by TP of paths).

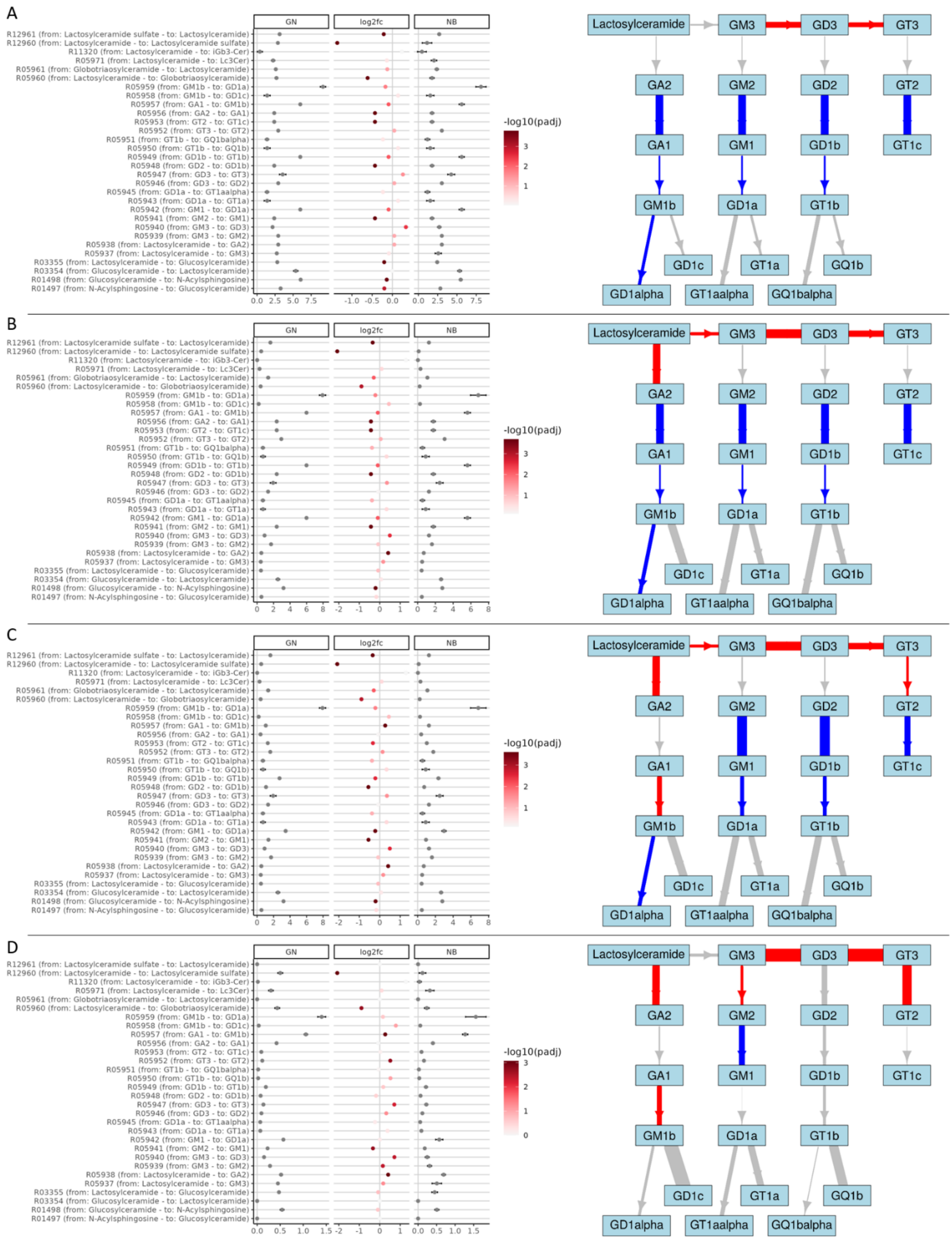

Fig. S3 Comparison of RAS-adjusted methods on the GSL pathway between NB and GN. Left dot plots illustrate the adjusted RAS values of NB and GN, as well as the log2 fold-change between the two groups. The whiskers indicate the standard deviation. The color intensity of log2 fold-change points indicates the negative decimal logarithm of the adjusted p-values. On the right, the subgraph containing the GSL pathway is visualized. Nodes represent the gangliosides and edges are the metabolic reactions (arrows). The arrow color describes the log2 fold-change direction, where red means that the reaction is more active in NB compared to GN and blue means the reaction is stronger in GN compared to NB. Gray arrows represent reactions that are not significant (adjusted p-value > 0.05). The thickness of the arrow represents the relative log2 fold-change. The subplots show A) values from M_W (RAS values), B) M_{W1} (RAS values adjusted by transition probability), C) M_{W2} (RAS adjusted by the recursive transition probability), and D) M_{W3} (RAS adjusted by transition probability of paths).

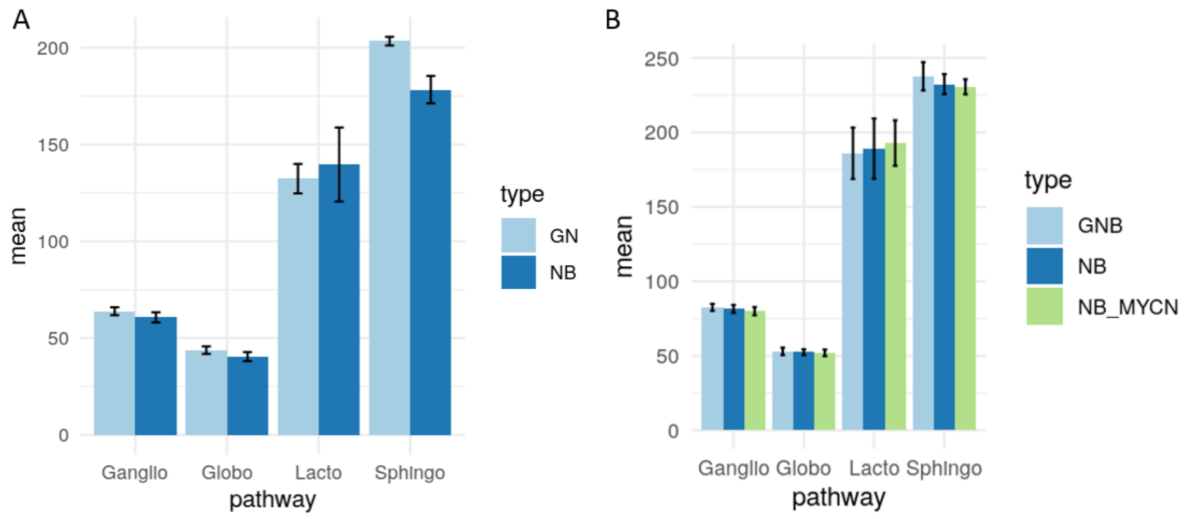

Fig. S4 Activity score per pathway. The mean activity score of a pathway is computed as the mean value of all sums of RAS reaction values associated with the respective pathway per sample. The whiskers show the standard deviation. The x-axis shows the four KEGG pathways. Ganglio = "Glycosphingolipid biosynthesis - ganglio series"; Globo = "Glycosphingolipid biosynthesis – globo series"; Lacto = "Glycosphingolipid biosynthesis - lacto and neolacto series"; Sphingo = "Sphingolipid metabolism"; NB = Neuroblastoma (MYCN not amplified); NB_MYCN = Neuroblastoma (MYCNamplified); GN = Ganglioneuroma; GNB = Ganglioneuroblastoma;

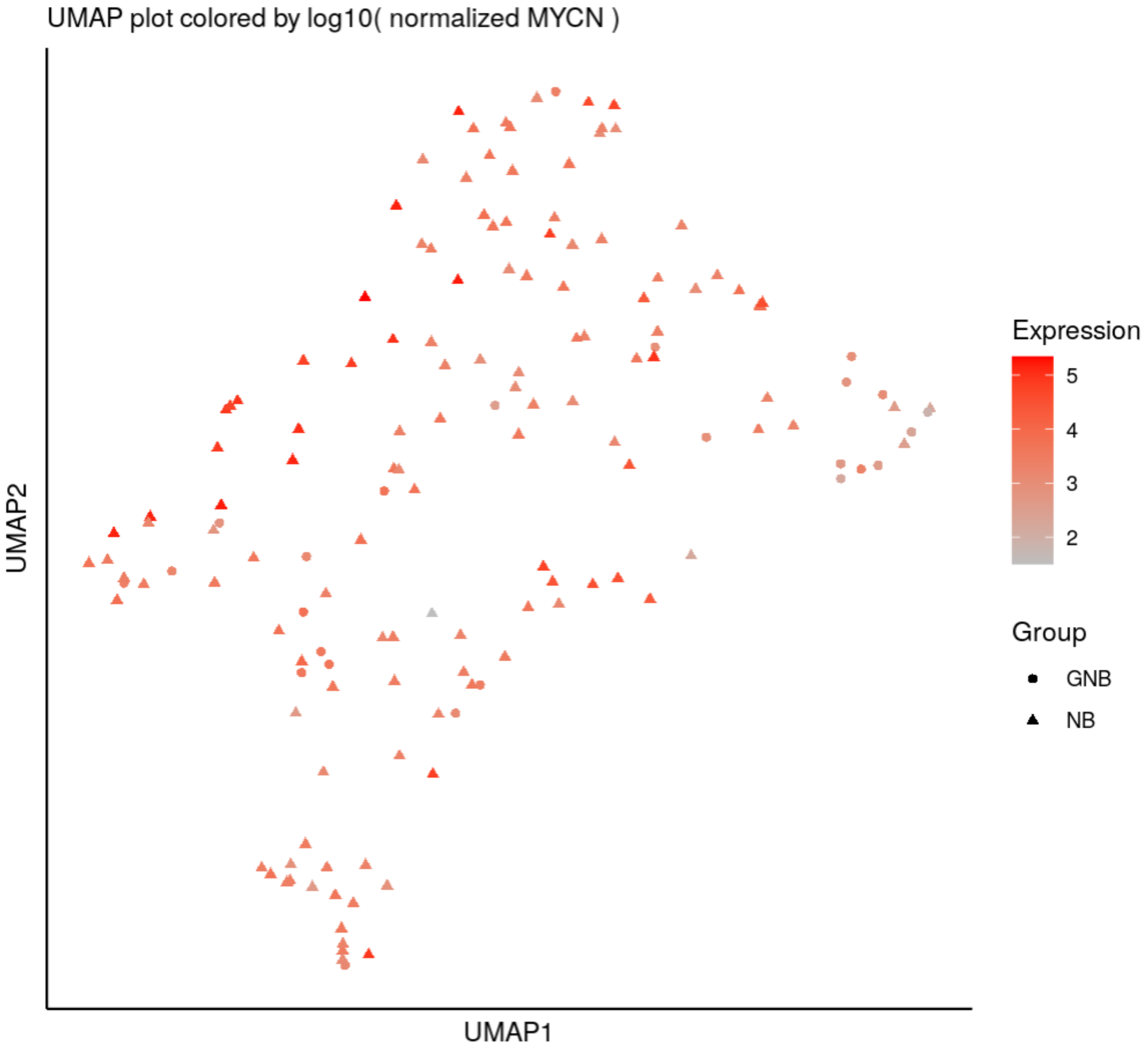

Fig. S5 UMAP representation (from Figure 10) was computed on RAS values of the NB/GNB TARGET dataset and colored by log10 normalized MYCN gene expression. Round symbols represent GN and triangles are NB samples.

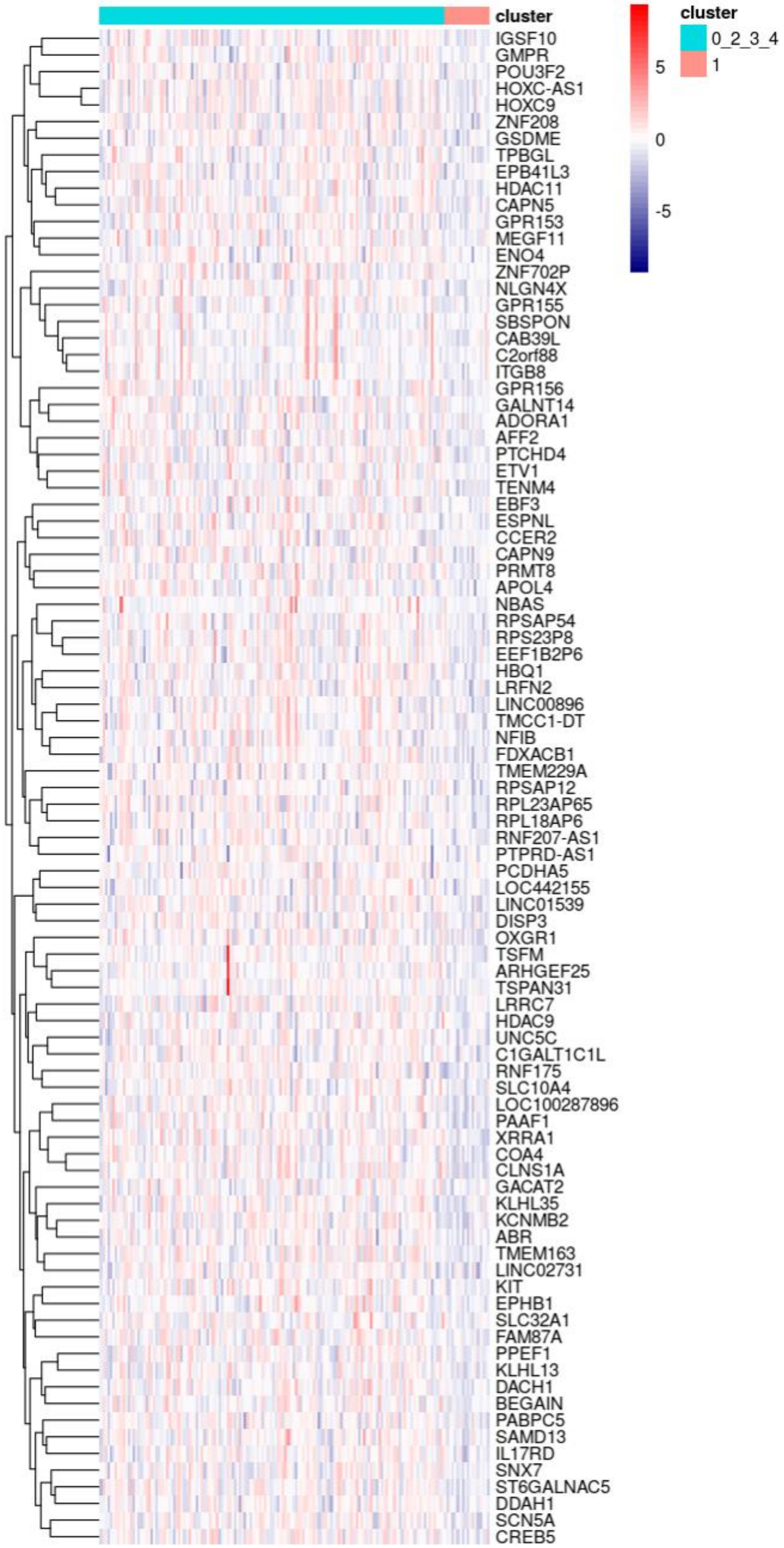
cluster
cluster
0_2_3_4
1
5
0
-5
IGSF10
GMPR
POU3F2
HOXC-AS1
HOXC9
ZNF208
GSDME
TPBGL
EPB41L3
HDAC11
CAPN5
GPR153
MEGF11
ENO4
ZNF702P
NLGN4X
GPR155
SBSPON
CAB39L
C2orf88
ITGB8
GPR156
GALNT14
ADORA1
AFF2
PTCHD4
ETV1
TENM4
EBF3
ESPNL
CCER2
CAPN9
PRMT8
APOL4
NBAS
RPSAP54
RPS23P8
EEF1B2P6
HBQ1
LRFN2
LINC00896
TMCC1-DT
NFIB
FDXACB1
TMEM229A
RPSAP12
RPL23AP65
RPL18AP6
RNF207-AS1
PTPRD-AS1
PCDHA5
LOC442155
LINC01539
DISP3
OXGR1
TSFM
ARHGEF25
TSPAN31
LRRC7
HDAC9
UNC5C
C1GALT1C1L
RNF175
SLC10A4
LOC100287896
PAAF1
XRRA1
COA4
CLNS1A
GACAT2
KLHL35
KCNMB2
ABR
TMEM163
LINC02731
KIT
EPHB1
SLC32A1
FAM87A
PPEF1
KLHL13
DACH1
BEGAIN
PABPC5
SAMD13
IL17RD
SNX7
ST6GALNAC5
DDAH1
SCN5A
CREB5

Fig. S6 Heatmap of downregulated genes in cluster 1 samples compared to all other samples.

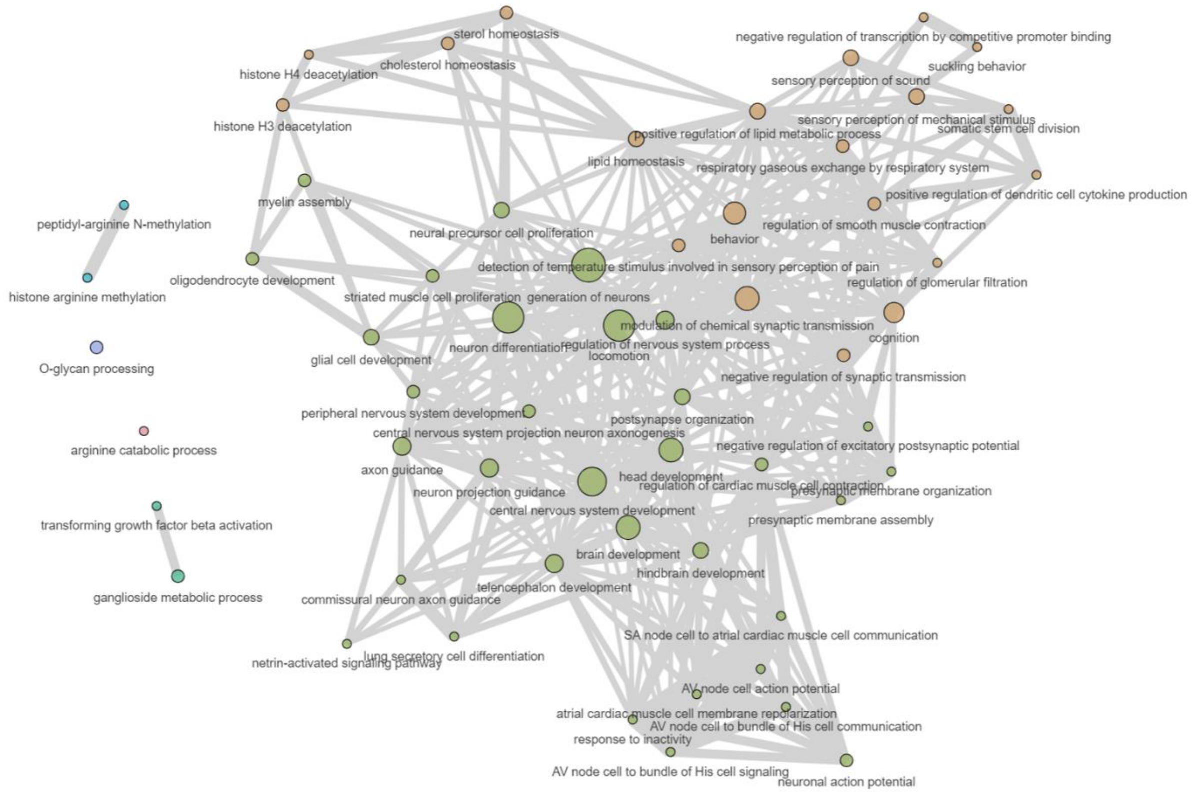

Fig. S7 Enrichment map of enriched GO terms based on downregulated genes in cluster 1 samples compared to all remaining clusters. The color of nodes indicates the assignment to a new meta geneset. Two big communities were identified involving 1) neuron and nervous system related terms (green), and 2) lipid-related and regulatory terms (brown). Edges indicate the degree of similarity (thickness) between the related GO terms above a threshold. The number of genes identified as differentially expressed is encoded in the node's size.

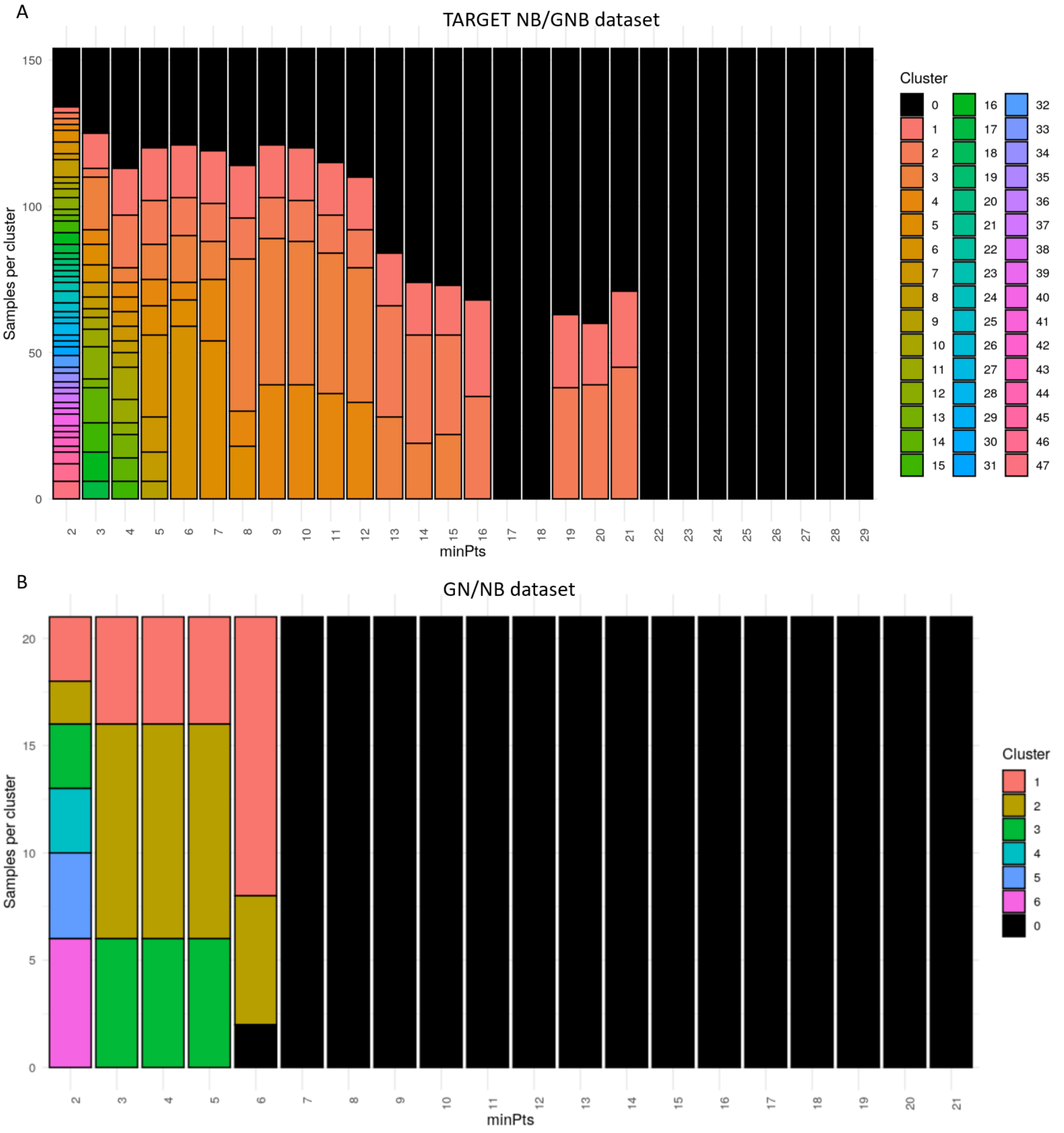

Fig. S8 Diagnostic plot for the optimization of the minimum cluster size parameter *minPts*. The number of samples per cluster and number of outliers (cluster 0) are visualized for different *minPts* values. This diagnostic plot was calculated based on the unadjusted RAS UMAP layout in A) TARGET NB/GNB dataset, and B) GN/NB dataset.

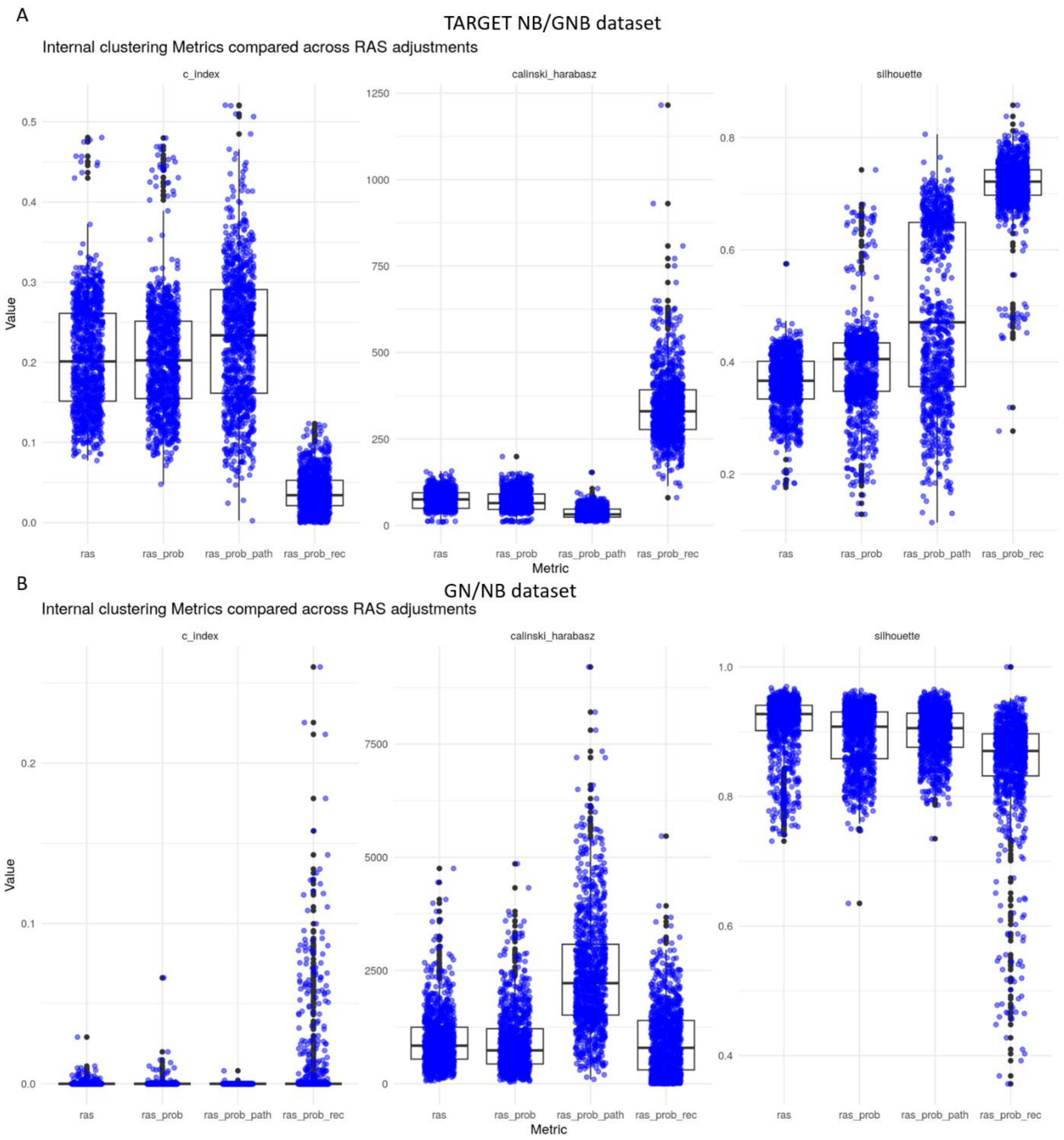

Fig. S9 Diagnostic plot of cluster consistency and separation across 1000 iterations per RAS adjustment. The intern metrics C-index, Calinski-Harabasz index, and silhouette score are used. A) TARGET NB/GNB dataset, and B) GN/NB dataset.
